# Longitudinal infant fNIRS channel-space analyses are robust to variability parameters at the group-level: An image reconstruction investigation

**DOI:** 10.1016/j.neuroimage.2021.118068

**Published:** 2021-08-15

**Authors:** Liam H. Collins-Jones, Robert J. Cooper, Chiara Bulgarelli, Anna Blasi, Laura Katus, Samantha McCann, Luke Mason, Ebrima Mbye, Ebou Touray, Mohammed Ceesay, Sophie E. Moore, Sarah Lloyd-Fox, Clare E. Elwell, BRIGHT Study Team

**Affiliations:** a Department of Medical Physics and Biomedical Engineering, University College London, London WC1E 6BT, UK; b DOT-HUB, Department of Medical Physics and Biomedical Engineering, University College London, London WC1E 6BT, UK; c Centre for Family Research, University of Cambridge, Cambridge, UK; d Department of Psychology, University of Cambridge, Cambridge, UK; e Department of Women and Children's Health, Kings College London, London, UK; f Centre for Brain and Cognitive Development, Birkbeck College, London, UK; g MRC Unit The Gambia at the London School of Hygiene and Tropical Medicine, UK

**Keywords:** Functional near-infrared spectroscopy, Image reconstruction, Infant functional neuroimaging, Infant cognitive development, Neurodevelopment, Longitudinal imaging

## Abstract

The first 1000 days from conception to two-years of age are a critical period in brain development, and there is an increasing drive for developing technologies to help advance our understanding of neurodevelopmental processes during this time. Functional near-infrared spectroscopy (fNIRS) has enabled longitudinal infant brain function to be studied in a multitude of settings. Conventional fNIRS analyses tend to occur in the channel-space, where data from equivalent channels across individuals are combined, which implicitly assumes that head size and source-detector positions (i.e. array position) on the scalp are constant across individuals. The validity of such assumptions in longitudinal infant fNIRS analyses, where head growth is most rapid, has not previously been investigated. We employed an image reconstruction approach to analyse fNIRS data collected from a longitudinal cohort of infants in The Gambia aged 5- to 12-months. This enabled us to investigate the effect of variability in both head size and array position on the anatomical and statistical inferences drawn from the data at both the group- and the individual-level. We also sought to investigate the impact of group size on inferences drawn from the data. We found that variability in array position was the driving factor between differing inferences drawn from the data at both the individual- and group-level, but its effect was weakened as group size increased towards the full cohort size (*N =* 53 at 5-months, *N =* 40 at 8-months and *N* = 45 at 12-months). We conclude that, at the group sizes in our dataset, group-level channel-space analysis of longitudinal infant fNIRS data is robust to assumptions about head size and array position given the variability in these parameters in our dataset. These findings support a more widespread use of image reconstruction techniques in longitudinal infant fNIRS studies.

## Highlights

• First investigation of validity of longitudinal infant channel-space fNIRS analysis.
• Novel image reconstruction analysis conducted.
• Variability in array position is dominant factor driving different inferences.
• Channel-space fNIRS analyses robust to implicit assumptions at group-level.
• Hope to encourage more widespread use of image reconstruction in infant analyses.

## 1 Introduction

The period of the first thousand days of life – from conception to 2 years of age – is a critical stage in the development of the brain and nervous system (Bornstein, 2014; Cusick and Georgieff, 2012; Mendez and Adair, 1999; Powell et al., 1995). The past twenty years have seen the adoption and optimisation of neuroimaging methods to further our understanding of development during this integral period of human life. However, while longitudinal studies of brain function play an important role in understanding development, less than a third of developmental neuroimaging studies published between 2008 and 2019 employed this design (Azhari et al., 2020).

While there has been an overall decreasing trend in published neuroimaging infant studies over the past decade (Azhari et al., 2020), recently and conversely there has been an increase in the number of studies employing functional near-infrared spectroscopy. This technique, abbreviated to fNIRS, is a non-invasive optical neuroimaging technique measuring changes in cortical haemoglobin concentration as a marker of functional activation (Lloyd-Fox et al., 2010; Pinti et al., 2020). Furthermore, there has been an increase in the use of this method in longitudinal studies of functional activation (Ichikawa et al., 2019; McDonald et al., 2019; Miguel et al., 2019) and functional connectivity (Bulgarelli et al., 2020b).

Longitudinal study designs are of particular importance when it comes to understanding the impact of early adversity on brain and cognitive development, and is a framework commonly adopted in global health projects. Most recently, fNIRS has found particular application in global health projects where the portability, low cost relative to functional magnetic resonance imaging (fMRI), and accessibility of the technology has enabled studies to be undertaken in low-resource settings (Blasi et al., 2019). Recent examples include studies of visual working memory in rural India (Wijeakumar et al., 2019); social selectivity in urban Bangladesh (Perdue et al., 2019); social markers in rural Gambia (Blasi et al., 2014; Lloyd-Fox et al., 2017); and monitoring treatment of malnutrition in infants and children in Guinea-Bissau (Roberts et al., 2017, 2020).

However, given this recent increase in the number of longitudinal developmental studies, particularly within the new frontier of global health neuroimaging research, it is paramount that we utilise analytical approaches that are applicable across a range of contexts (such as age and changes in head size). In fNIRS, an array of sources and detectors are placed on the head. Each detector records the intensity of light arriving from a subset of neighbouring sources, with each dual-wavelength source and detector pair referred to as a channel. Typically, the analysis of fNIRS data occurs in the channel-space, where data from each channel is pre-processed and statistically examined on a channel-by-channel basis. Group-level channel-space analyses are then predicated on the notion that data acquired from the same channel of the same array can be compared between (and combined across) individuals. This approach makes two assumptions. The first is that **differences in scalp positions of sources and detectors relative to cranial landmarks are negligible across individuals**. The second is that **a given scalp location has the same spatial relation to underlying cortical anatomy across all individuals**. This is particularly pertinent in longitudinal studies over the first year of life, where head circumference increases by almost a quarter from 1- to 12-months of age (World Health Organization, 2007). This issue concerns not only scalp-cortex correspondences, but also differences in cortical depth linked to variation in head size.

To produce images from fNIRS data, an image reconstruction approach can be used. Image reconstruction employs a structural prior of head anatomy to compute a forward model of the propagation of near-infrared light, describing how an attenuation change at a given point in the head will affect resulting fNIRS attenuation measurements. This model is then mathematically inverted, and optical density data derived from fNIRS attenuation measurements for each channel is combined with the inverted forward model to reconstruct an image that maps cortical haemoglobin concentration changes (Arridge and Cooper, 2015).

Channel-space analyses assume a constant head size and a constant array position across participants. Variability in either of these parameters will influence the distribution of near-infrared light transmitted from source to detector, and will therefore influence measures of brain activation. Here, we aim to provide an analysis to isolate the effects of the variability in head size and array position on the analysis of longitudinally-acquired infant fNIRS data. Because these effects are fundamentally related to the three-dimensional anatomy of the subject, such an analysis requires a light transport modelling and image reconstruction approach. Using such an approach, we can directly compare the effects of head size and array position in a consistent anatomical space. We therefore chose to implement an image reconstruction approach to isolate the effects of variability in these parameters, which we can then use to infer the effects of variability in these parameters on conclusions about fNIRS data analysis drawn from channel-space analyses.

In this work, we use image reconstruction as a tool to investigate the validity of assuming constant array position and constant head size in channel-space analysis of longitudinal infant fNIRS data. Specifically, this paper:-

1 investigates whether the application of a best-practice image reconstruction approach can result in different statistical inferences compared to a standard channel-space analysis.
2 uses image reconstruction approaches to investigate the effect of variation in array position and head size on the interpretation of fNIRS data.
3 uses image reconstruction approaches to investigate whether group size has an impact on differing statistical and anatomical inferences between a best-practice image reconstruction approach and an image-space equivalent to channel-space analysis.

To address these objectives, this paper utilises data from the Brain Imaging for Global Health (BRIGHT) project. This is a longitudinal study investigating early neurocognitive development during the first 2 years of life, following two cohorts of infants in parallel; one in The Gambia (*N =* 225) and the other in the UK (*N =* 62). As part of the BRIGHT project, fNIRS data was collected at six age points: 1-, 5-, 8-, 12-, 18- and 24-months of age. Data from this project has already been analysed to investigate age-related changes in the neural responses to tasks such as assessing working memory (Begus et al., 2016), social cognition (Lloyd-Fox et al., 2017, 2014a), and habituation and novelty detection (Lloyd-Fox et al., 2019). Due to its large sample size and its inclusion of data acquired at three age points up to 12-months of age, the dataset from The Gambian cohort of the BRIGHT project is highly suited to address the objectives outlined above.

## 2 Methods

### 2.1 Participants

Recruitment of participant families occurred at the Medical Research Council (MRC) Unit The Gambia at the London School of Hygiene and Tropical Medicine (MRCG@LSHTM) field station in Keneba, The Gambia, during antenatal clinic visits. In order to avoid confounds relating to language translation, only families of the Mandinka group, the ethnic majority in the region (Hennig et al., 2017), were recruited. All infants included in the current study were required to have been born at term (37–42 weeks gestation). Datasets from 104 infants aged 5-months, 97 infants aged 8-months, and 97 infants aged 12-months in the Gambian cohort of the BRIGHT study were available at the time of conducting this analysis. Only datasets from the Gambian cohort were included in this analysis. This was because the Gambian cohort had a particularly large sample size, particularly in the context of longitudinal infant studies. Furthermore, for this analysis we wanted to avoid the potential confounds related to cohort demographics that would come by mixing data from the two cohorts.

In the West Kiang District, where Keneba is situated, moderate to severe growth faltering is prevalent in infants from roughly 3 months of age, due to several factors which include prenatal growth retardation, poor-quality (often contaminated) foods and a high incidence of infection (Lunn et al., 1991; Lunn, 2000; van der Merwe et al., 2013)). As part of the BRIGHT project, growth measurers were acquired at each age point, though an indication of severe growth faltering (i.e. weight‐for‐height z-score or head circumference z‐score greater than 3 below the median values stated in the World Health Organisation standards (World Health Organization, 2007)) was not a criteria for exclusion.

Ethics approval for the BRIGHT study was obtained from the joint Gambia Government/MRC Unit The Gambia Ethics Committee (‘Developing brain function for age curves from birth using novel biomarkers of neurocognitive function’, SCC number 1451v2). Full, informed consent was obtained from all participating families prior to recruitment.

### 2.2 Procedure

**Fig. 1:**
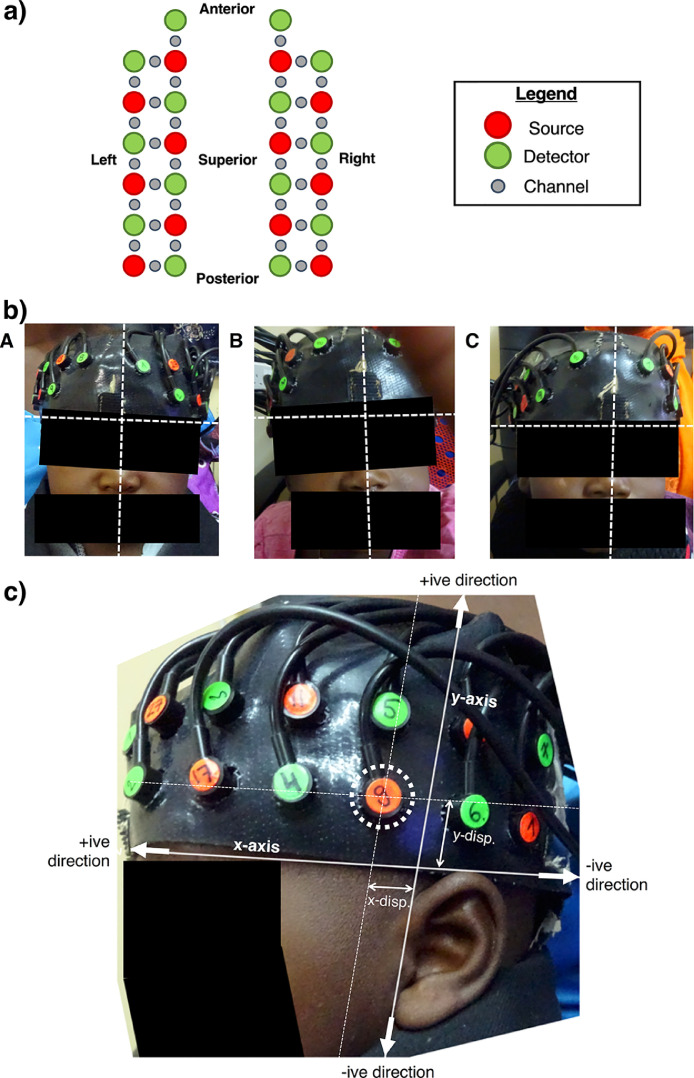
a) Representation of the BRIGHT array, outlining the positions of sources and detectors (see legend). b) Anterior headgear placement of three infants included in the study. The horizontal dotted line denotes the level of the top of the eyebrows, and the vertical dotted line denotes the midline. A: vertical line denoting middle of the headband is uncentered relative to the midline, but the bottom of the headband is not displaced relative to the top of the eyebrows. B: bottom of headband is displaced superiorly with respect to the top of the eyebrows, but is centred relative to the midline. C: headgear is centred relative to the midline and is in line with the top of the eyebrows. c) Lateral assessment of headgear placement. The displacement of a reference optode, highlighted by a dotted circled, in directions parallel to the *x*- and *y*-axes is measured (denoted by “x-disp.” and “y-disp.”). Displacement in the anterior or superior directions were taken to be positive, while displacement in the posterior or inferior directions were taken to be negative.

For fNIRS data acquisition, infants wore custom‐built fNIRS headgear consisting of two arrays, one over each of the left and right hemispheres, embedded within a custom-made soft silicone-based head band. The arrays contained a total of 6 sources and 7 detectors per hemisphere (source‐detector separations 2 cm), constituting 17 channels per hemisphere (Fig. 1a). Data were acquired with the NTS fNIRS system (Gowerlabs Ltd. London, UK) which uses two continuous wavelengths of light at 780 nm and 850 nm and has a sampling rate of 10 Hz (Everdell et al., 2005). The design of the fNIRS array enabled responses in lateral frontal to posterior temporal brain regions to be investigated, which included the inferior frontal gyrus; middle and superior temporal regions; and the temporo‐parietal junction.

Any displacement of the headband that could compromise the stability of its fit were excluded from further analyses. To assess headgear placement, photographs of the array were taken on each participant's head pre- and post-experiment. Over the anterior of the head, the intended placement of the headgear should align a vertical line denoting the centre of the band to the midline (in line with the participant's nasion landmark) and such that the silicone band lay just above and in line with the eyebrows. Infants where the headband was displaced both horizontally (with reference to the midline) and vertically (with reference to the eyebrows) were excluded from further analyses on the grounds of poor placement of headgear. Examples of three included infants with horizontal but no vertical displacement (A), vertical but no horizontal displacement (B), and no horizontal or vertical displacement (C) over the anterior of the head are shown in Fig. 1b.

The placement of the headgear was assessed laterally by overlaying a set of axes on the images of the head to quantify the displacement of a reference optode. This method was first demonstrated by Blasi et al. (2014). The intended placement of the headgear was such that the third lower optode from the posterior, used as a reference optode (see Fig. 1c), was over the tragus. The overlaid *x*-axis was defined as a line from the top of the eyebrows running along the superior-most point of the ear, while the *y*-axis was defined as a line passing through the tragus and the anterior of the helix. Displacement in the directions of both *x*- and *y*-axes were measured; displacement towards the anterior or superior direction was noted as positive, while displacement towards the posterior or inferior direction was noted as negative. The x-displacement of the reference optode was measured, while the vertical displacement of the bottom headband at that x-displacement value was measured and then added to the value of the lower band thickness to obtain the y-displacement. The x- and y-displacement values for each hemisphere for each individual were used to register the array to a head model. If the x-displacement value was greater than or equal to 1.6 cm, channels were re-indexed such that the array was shifted either forward or backward one full channel space. If the y-displacement was greater than 1.6 cm, the infant was excluded from further analyses on the grounds of poor headgear placement.

In addition, head circumference, tragus-to-tragus distance and nasion-to-inion distance were measured for each participating infant. Infants without a head circumference measurement were excluded.

### 2.3 Experimental paradigm

Participants were assessed using an auditory-visual social perception paradigm first described by Lloyd-Fox et al. (2014a) and subsequently used in a number of other studies of infant brain function (Frijia et al., 2020; Lloyd-Fox et al., 2017, 2013, 2012, 2018; Perdue et al., 2019).

The paradigm included three experimental conditions and a baseline condition. During each condition, visual-social videos were presented, showing Gambian adults moving their eyes left or right, or performing hand games. The duration of these videos ranged from 9 to 12 s. In the *visual-social silent* (VS) condition, visual-social videos were presented in silence with no accompanying audio. At the onset of two in every three trials, auditory stimuli were presented, lasting a total duration of 8 s (consisting of four different sounds). The *auditory vocal* (V) condition was where infants were presented with non-speech vocalisations of two adult speakers (who were either coughing, crying, laughing or yawning) alongside the visual-social videos. The *auditory non-vocal* (NV) condition was where common environmental sounds familiar to the infants that were not human- or animal-generated were presented alongside the visual-social videos.

Experimental conditions were altered one after the other, and the same order of conditions (VS, NV, V, VS, V, NV) was presented until the infant showed signs of fussiness or boredom or up to the point that 5 presentations of each condition has been reached. During fNIRS data acquisition, videos of the infants were recorded to perform eye-tracking to monitor the time the infant was looking at the screen for each trial, which was used as an indication of the infant's attention. A baseline condition was presented between experimental conditions, where images of types of transport (such as helicopters, cars, and trains) were displayed. A graphical representation of the paradigm is shown in *Supplementary Material*.

In this work, we focused on the response to the auditory vocal stimulus. Usually, the response to this condition is studied in the context of its contrast with the response to the auditory non-vocal condition. However, here we aimed to investigate whether different data analysis approaches (in both image- and channel-space) can lead to different inferences drawn from the data rather than the contrast between the two conditions. As such, we chose to focus on the response to a single condition.

### 2.4 fNIRS data pre-processing

The fNIRS data were pre-processed using NirsPlot (Hernandez and Pollonini, 2020) and Homer2 (MGH–Martinos Center for Biomedical Imaging, Boston, MA, USA) (Huppert et al., 2009), which were both implemented in MATLAB. The specific pipeline and analysis have previously been reported by Bulgarelli et al. (2020a)) and Hernandez and Pollonini (2020).

The first step in the processing stream was channel pruning. Based on previous experience with the NTS fNIRS system, channels with intensity readings lower than a certain threshold were immediately excluded. The data were then subjected to a cardiac-signal and spectral analysis assessment based on a method first proposed by Pollonini et al. (2016). This inspection was done within each channel in the array. Channels that did not pass these quality assessments were excluded from further analysis. If more than 40% of channels from a given dataset were deemed invalid, then the whole dataset was excluded from further analysis.

For each of the four conditions, raw intensity data from surviving channels were processed in a pipeline in Homer2. The first step converted raw intensity data to optical density. Motion artifacts were corrected using a combination of spline interpolation and wavelet-based filtering in the method proposed by Di Lorenzo et al. (2019). Following motion artifact correction, sections of the data still affected by noise were flagged: if such an artifact was detected, a time window wider than the extent of the flagged section was defined on either side of the artifact. Trials within this time window were excluded from the analysis, where trial exclusion was applied within each channel. The data were band-pass filtered with high- and low-pass frequencies of 0.02 and 0.06 Hz respectively in order to correct for slow baseline drifts in the data as well as to eliminate high-frequency noise. The modified Beer-Lambert law was employed to convert optical density data into concentration changes in oxy- and deoxy-haemoglobin (Delpy and Cope, 1997). The differential pathlength factors were calculated for each wavelength and age using the formula proposed by Scholkmann and Wolf (2013).

Based on the looking time measures, trials where the infant was looking at the screen for less than 60% of the trial's duration were excluded, and infants with less than a minimum of three valid trials for the auditory vocal condition were excluded. In Homer2, all the remaining trials (after exclusion for looking time and excessive noise due to motion) for each participant were block-averaged for each condition: the block duration was defined starting at *t* = −2 s from stimulus onset and ending at *t* = 20 s from stimulus onset. The total duration of the block was therefore 22 s.

As previously mentioned, though data was pre-processed for all four conditions, we only focus on the response to the auditory-vocal stimulus relative to baseline.

### 2.5 Processing streams

In this work, five data processing streams were used: channel-space analysis and four image reconstruction pipelines. In the channel-space analysis, the block-averaged values of concentration changes across infants during a response window were compared to baseline for the auditory vocal condition. For the image reconstruction processing stream, each individual's block-averaged concentration changes were converted back to optical density values using the modified Beer-Lambert law. The optical density values were then used as an input in the image reconstruction step.

The basic outline for each of the imaging processing streams was as follows:

1 Warp a head model on the basis of certain head measurements.
2 Register optode positions to the head model.
3 Produce a forward model, which defines the sensitivity distribution associated with each channel.
4 Invert the forward model, and use as an input alongside a given infant's block-averaged optical density data into a reconstruction function to produce a time-course image of the distribution of haemoglobin concentration changes on the cortical surface.
5 Repeat steps 1–4 for all participants.
6 Perform group-level statistical analysis on a node-by-node basis, comparing the concentration change values across participants in a response window to baseline.

**Fig. 2:**
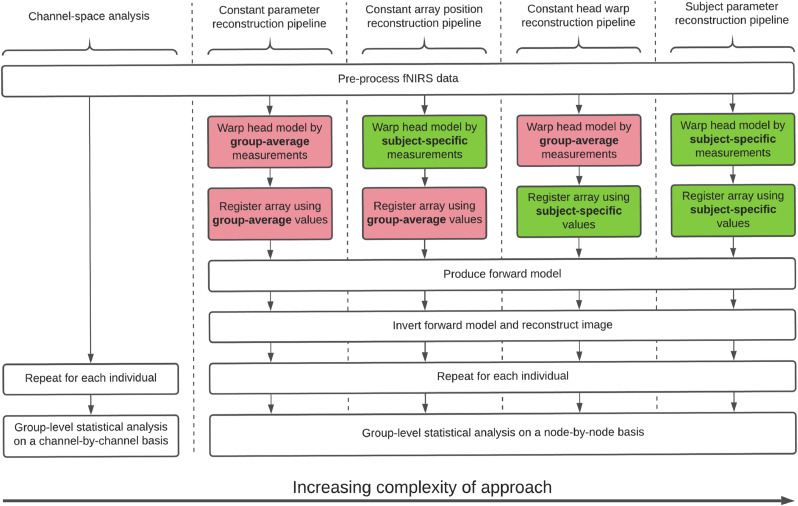
Outline of the different processing streams compared in this study.

For step 1, the head model could be warped on the basis of either (a) subject-specific head measurements, or (b) group-average head measurements. For Step 2, there were two options for registering the array to the head model: (a) register by subject-specific array positioning data, or (b) register by group-average array positioning data. Given these two steps in the processing pipeline, each with two options, there were a total of four possible image reconstruction processing streams. All four of these processing streams are outlined in Fig. 2.

For the purpose of this analysis, the first processing stream presented, where subject-specific head measurement data were used both to warp the head model and to register the array, is termed *subject parameter reconstruction*. Given that this processing stream attempted to account for array position and head size on a subject-specific basis, this was effectively the best-practice image reconstruction pipeline that could feasibly be applied to this dataset. At the other end of the complexity scale, the second processing stream used group-average head measurement data to warp the head model and group-average array position data to register the array, and is termed *constant parameter reconstruction*. This processing stream employed the same assumptions as the channel-space analysis, and is considered as an image-space equivalent to channel-space analysis. While some fNIRS analyses may make not attempt to account for head size, we believe a reasonable minimum step for research groups to take is to obtain a population average measure of head size and use that measurement to warp a head model for each age group. We use this approach to define our constant parameter pipeline, which provides a reasonable baseline against which to compare our subject parameter pipeline.

Two other variations of the image reconstruction pipeline are compared, both attempting to isolate the effects of variability in head size and array position. *Constant array position reconstruction* (subject-specific head measurements, group-average array position) offered a method to isolate the effect of variability in array position, and *constant head warp reconstruction* (group-average head measurements, subject-specific array positioning) offered a method to isolate the effect of variability in head size.

We acknowledge that some fNIRS pipelines may not account for head size at all. We therefore re-ran our constant parameter pipeline twice for the fNIRS data at each age, using the head measurements of the other ages, to mimic the case where a single-sized head model is used for all infants.

### 2.6 Head modelling

A four-layer mesh model of the infant head was used as part of the image reconstruction process, which was constructed using structural MRI data from a cohort of 12-month-old infants presented by Shi et al. (2011). A single head model was used across ages and spatially warped appropriately. Prior to choosing to employ a single head model, we conducted an extensive preliminary analysis to evaluate age-specific models for the age range investigated in this work using structural data presented by Sanchez et al. (2012). This analysis demonstrated very little differences in sensitivity as a result of anatomical differences (i.e. spatial distribution of tissues but not model size) across these models, and is presented in *Supplementary Material*. Using a single model across ages also negated the need to register different head models to a common space for comparison across ages, which would undoubtedly have incurred some level of error itself.

Binary tissue masks for white matter, grey matter and cerebrospinal fluid were combined to produce a cerebral tissue mask. The outer boundary of the cerebral tissue mask was used to demarcate the inner skull border, and an outer scalp boundary was segmented from the average T1-weighted MRI template using Betsurf (Jenkinson et al., 2005). All voxels between the outer scalp and inner skull boundaries were defined as extra-cerebral tissue, a combined layer for scalp and skull; given the difficulty involved in distinguishing the two tissues from each other in infant MRI data, the two tissues were combined as one label. The resulting four-layer tissue mask was used to create a tetrahedral volume mesh as well as a grey matter surface mesh using the iso2mesh package ((Fang and Boas, 2009), see iso2mesh.sourceforge.net).

A parcellation atlas, the Automated Anatomical Labelling (AAL) atlas, consisting of 90 volumes of interest, was transformed to the space of the 12-month head model based on the affine transformation information in its file header. This allowed us to use the AAL atlas to assign an anatomical label to each node in the grey matter surface mesh.

**Fig. 3:**
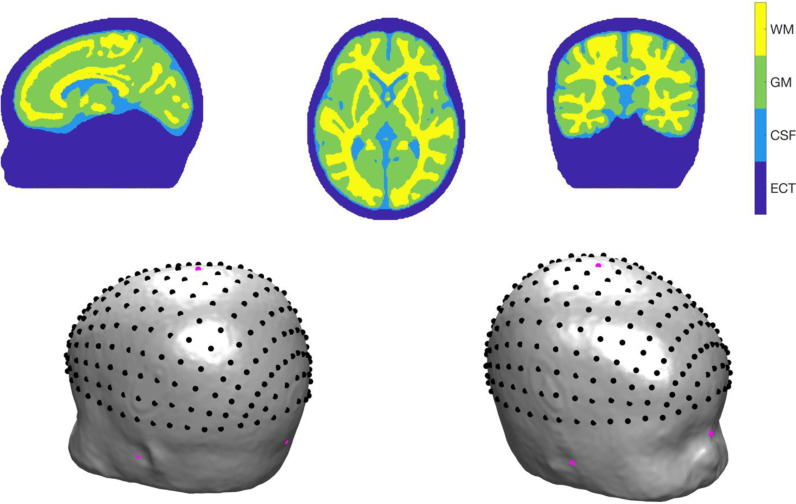
Top row: example sagittal, axial and coronal sections of the four-layer infant head model showing the distribution of white matter (WM), grey matter (GM), cerebrospinal fluid (CSF) and extra-cerebral tissue (ECT). Bottom row: the position of the cranial landmarks and 10-5 positions (in black) and cranial landmarks (in magenta) on the scalp surface.

The coordinates of five cranial landmarks (the nasion (Nz), the inion (Iz), the left pre-auricular point (Al), the right pre-auricular point (Ar) and vertex (Cz)) were determined manually using ITK-SNAP (Yushkevich and Gerig, 2017). Based on a curve-walk procedure ((Aasted et al., 2015), see Homer2: www.nitrc.org/projects/homer2), the 10-5 positions on the scalp surface of the head model were computed using the cranial landmarks coordinates (see Fig. 3). The mesh nodes were then transformed to a coordinate system where:

• the position of Iz defines the origin
• a line joining Iz to Nz defines the y-axis
• the z-coordinates of Ar and Al are approximately equal following rotation of mesh nodes around the y-axis.

### 2.7 Head model warping

Data on head circumference, tragus-to-tragus via Cz (approximated to be the Ar-Cz-Al) distance, and nasion-to-inion via Cz (Nz-Cz-Iz) distance were used to iteratively warp the head model. For the pipelines that required the use of subject-specific head measurements (subject parameter and constant array position pipelines), these measures were used to warp the infant head model to each participant's head dimensions. For the pipelines that did not require subject-specific head measurements (constant head warp and constant parameter pipelines), the mean values of these measures were calculated at each age and used to iteratively warp the infant head volume mesh. At the 5-month assessment timepoint, measurement of the Nz-Cz-Iz distance was not taken, and so the head model was warped according to head circumference and Ar-Cz-Al distance. The grey matter surface mesh was also iteratively warped by the same values as the head model.

For a given participant, the head model was initially scaled according to head circumference; a warp factor was calculated by dividing the subject-measured (or group mean) head circumference by the head model's initial head circumference. Each node's x-, y- and z-coordinates were multiplied by the warp factor. The warped model's head circumference, Ar-Cz-Al distance and Nz-Cz-Iz distance were computed, and the measurement with the greatest difference between its corresponding subject-measured (or group mean) value was then used to re-warp. For whichever distance had the greatest difference, the warped model-measured value was divided by the subject-measured (or group mean) value to yield another warp factor which was then multiplied by the relevant node coordinates (x- and z-coordinates if Ar-Cz-Al; y- and z-coordinates if Nz-Cz-Iz; x-, y- and z-coordinates if head circumference). The process was repeated until the error for the Ar-Cz-Al distance was below 6 mm, the error for the Nz-Cz-Iz distance was below 6 mm, and the error for head circumference was below 3 mm. See Supplementary Materials for details on accuracy of head model warping with respect to participant-measured values. Allowing a degree of tolerance with regards to head measurements (which themselves are prone to a degree of error) removes the potential for over-fitting the head warping procedure to head measurements, which can lead to anomalous and anatomically implausible head shapes. The parameters chosen were a balance between over-fitting, accuracy and computation time. In *Supplementary Material*, we provide data on the accuracy of our iterative warping procedure in preserving participant-measured head measurements in the warped head models for each infant undergoing the subject parameter pipeline. This error rarely exceeded 2% for Nz-Cz-Iz and Ar-Cz-Al, and rarely exceeded 0.2% for head circumference.

### 2.8 Array registration

For each hemisphere the approximated x-axis on the warped head model was defined as a curve along the scalp surface from Iz to FPz along the lateral side of the head, and the y-axis was approximated by defining a curve from the preauricular point to CCPz on the midline of the head. The x- and y-displacement values of the reference optode extracted from photographs were used to register the reference optode to the head model. With the knowledge of a source-detector separation of 2 cm between nearest neighbours, the other optodes in the array were registered to the head model in relation to the reference optode along two curves parallel to the Iz-FPz curve.

**Fig. 4:**
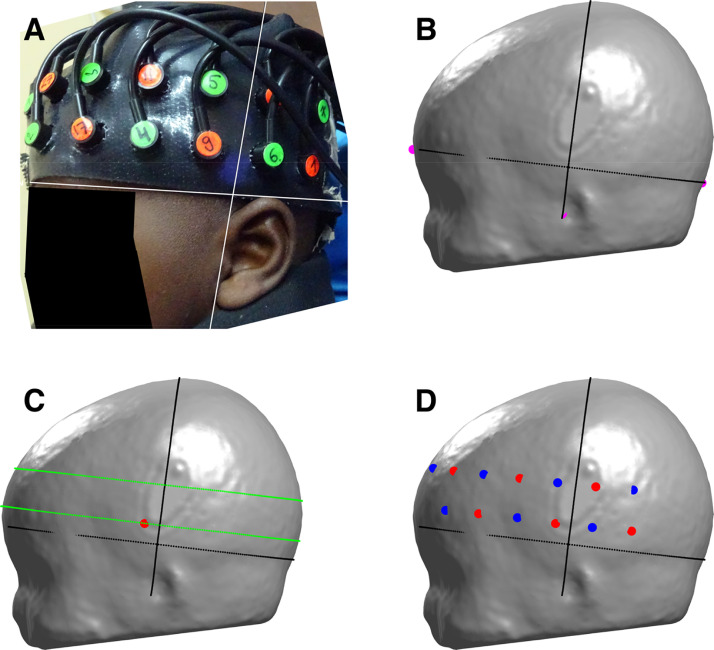
The array registration process for the subject parameter reconstruction pipeline. A) Photograph of the lateral placement of the array on an example infant, with the *x*- and *y*-axes overlaid. B) The *x*- and *y*-axes approximated on the head model warped on the basis of the infant's head measurements. C) Curves (in green) parallel to the Iz-FPz curve which were used to register optodes in relation to the reference optode, shown as a red circle. D) All optodes registered to the head model, where detectors are represented by blue circles and sources are represented by red circles.

For the subject parameter and constant head warp pipelines, subject-specific values were used to register optodes to the head model. For constant parameter and constant array position pipelines, age-cohort mean *x*- and *y*-axis displacement values were used. An example of a registered array on the head model compared to the corresponding participant photograph is provided in Fig. 4.

### 2.9 Light transport modelling and image reconstruction

For each infant dataset in each processing stream, we used TOAST++ ((Schweiger and Arridge, 2014), see http://toastplusplus.org) to model near-infrared light transport to produce a forward model for each wavelength. Using a regularization hyperparameter of 0.1, a zeroth-order Tikhonov regularized reconstruction was performed. A requirement for depth discrimination in image reconstruction is that overlapping channels are present in the array (Lee et al., 2017); these are channels that exhibit sensitivity profiles that partially sample the same volume (Boas et al., 2004; White, 2010). No data from overlapping channels were used in this analysis, and so image reconstruction was constrained to the grey matter nodes of the volume mesh. This has been shown in previous topographic approaches to increase the accuracy of reconstructed images when data on cortical activation is being collected (Boas and Dale, 2005; Boas et al., 2004). In addition, we do not expect to be sampling a substantial amount of white matter due to the source-detector separation in our array.

For each individual, the forward model was thresholded at 1% of the maximum value to produce a binary image, which was mapped to the grey matter surface mesh to create an individual-level grey matter mask. A group-level grey matter mask for each age-cohort was produced, which consisted of nodes present in the individual-level grey matter masks of at least three quarters of participants, similar to the approach taken by Wijeakumar et al. (2019). Data preparation, meshing, forward modelling and reconstruction were facilitated by the DOT-HUB Toolbox (www.github.com/DOT-HUB).

### 2.10 Statistical mapping of reconstructed images

In this work, we produced T-statistic maps comparing the group-level response to the auditory vocal condition to baseline across participants for a given age. Statistical mapping was conducted in the space of the grey mater surface mesh. A time window of 12–16 s post-stimulus onset was used to signify the peak of the haemodynamic response in the image time-course. This choice of window was informed by previous data analysis approaches using the same paradigm in previous cohorts (Lloyd-Fox et al., 2017, 2013, 2012).

Statistical mapping was performed for oxy-haemoglobin group-level images at each age and for each image reconstruction processing stream. We also present deoxy-haemoglobin group-level images for the subject parameter pipeline in the *Results* section. All concentration change values within the response window across participants were concatenated to produce a single vector for each node present in the group-level grey matter mask; the same process was completed for values within a baseline window (2 s pre-stimulus onset). Equivalent response and baseline vectors were compared using a two-tailed two-sample t-test. To correct for multiple comparisons, the Bonferroni method was employed on the basis of the number of nodes in the grey matter surface mesh, as was performed in (Frijia et al., 2020).

### 2.11 Statistical mapping of channel-space analysis

We conducted a channel-wise statistical analysis comparing the group-level response to the auditory vocal condition to baseline across all infants, in a method analogous to the statistical mapping approach for the imaging processing stream. Statistical analyses were performed separately for oxy- and deoxy-haemoglobin time-courses. To be included in the analysis for a given age cohort, the channel had to be present (i.e. not pruned) in at least three quarters of individuals. Using a 12–16 s time window to represent the peak of response and a 2 s pre-stimulus baseline, a concatenated vector of concentration change values in these periods in each channel across all infants were compared using a two-tailed two-sample *t*-test. To correct for multiple comparisons, the Bonferroni method was employed on the basis of the channels (34 in total).

To enable the group-level channel-space analysis to be compared to reconstructed images, channel location positions on the scalp were projected onto the cortical surface. It is commonly assumed in fNIRS work that the region of the brain to which a channel is maximally sensitive is halfway between the source and the detector, and at a depth from the scalp surface equal to approximately half the source-detector separation (Fukui et al., 2003; Lloyd-Fox et al., 2014b). Using this knowledge, previous work relating to fNIRS in infants has demonstrated cortical projection to determine the cortical label and position of channels (Lloyd-Fox et al., 2014b; Tsuzuki et al., 2017).

For each age, the head model was warped by the group-average head measurements and the group-average array positioning data was used to register optode positions. The midpoint on the scalp surface between source and detector for each channel was projected onto the cortex in a method analogous to that which was demonstrated in (Collins-Jones et al., 2020) which employs the Möller-Trumbore algorithm (Möller and Trumbore, 1997; Mena-Chalco, 2019).

The surface nodes of the volume mesh (i.e. the scalp surface) that were situated within a 5 mm radius of each source-detector midpoint were determined, and were used to fit a plane. Orthogonal to this plane, a vector was defined whose length was increased until it intersected with a face on the grey matter surface mesh. The position of this intersection was taken to be the cortical projection of the channel.

### 2.12 Window-averaged images

For each individual, the duration of the block-averaged pre-processed data for the auditory vocal condition for each channel is 22 s (consisting of a 2 s pre-stimulus onset baseline period plus a 20 s post-stimulus onset period), and so each infant's reconstructed image is a time-course of 221 frames. To obtain a single image for a given individual at a given age point, the mean value of each grey matter surface mesh node in a 12–16 s window post-stimulus onset was computed, to yield what we term a *window-averaged image*.

### 2.13 Metric extraction

In order to compare images between processing streams, two metrics were used: *peak node offset* and *cortical label of peak node*, which were computed separately for both left and right hemispheres. For subject parameter and constant parameter images at the group-level, the peak node was defined as the node in the group-level image with the greatest positive and negative T-statistic value for oxy- and deoxy-haemoglobin concentration changes, respectively. In addition, the cortical label of the peak node was determined. This was completed for both hemispheres at each age.

For channel-space analyses, the peak channel was defined as the channel with the greatest positive and negative T-statistic value for oxy- and deoxy-haemoglobin concentration changes, respectively. To enable comparison between channel-space analysis and subject parameter reconstruction, the cortical label of the peak channel projection was determined and used as an analogous metric to the cortical label of peak node. This was completed for both hemispheres at each age.

At the individual-level, the peak node was defined as the node in the window-averaged image with the greatest positive change in oxy-haemoglobin concentration. This was completed for both hemispheres for each infant at each age. We focus only on changes in oxy-haemoglobin concentration due its larger response than deoxy-haemoglobin, which was an important consideration given the lower signal-to-noise ratio in the individual-level images.

At both the group- and the individual-level, we defined the peak node offset as the Euclidean separation between the peak node from a given processing stream and the peak node from the subject parameter reconstruction. This was calculated in the space of the relevant age-cohort constant head warp model. In addition, having determined the peak node, the cortical label of that node was identified using the parcellation obtained using the methods presented in Section 2.6 “Head modelling”.

### 2.14 Effect of longitudinal growth measures

As part of our investigation on the effect of head size, we sought to investigate whether there was any association between peak node offset and two other parameters: head circumference (as measured from the infants, not corrected for age and sex) and head growth trajectory (the change in head circumference z-score between two age points). The purpose of this comparison was to assess whether the use of subject-specific parameters was more impactful in infants whose head size deviated from the group mean at a given age or whose head growth trajectories diverged from the expected trajectories outlined by the World Health Organisation (WHO) growth charts (World Health Organization, 2007). This is important to investigate as differences in head size and growth trajectory could potentially lead to notable differences in light transport through the head which may lead to artifactual statistical or anatomical inferences.

For each individual, the difference in head circumference from the group mean was calculated. For each age and each hemisphere, using Pearson correlation we tested for associations between individual-level peak node offset and:-

1 Difference in head circumference from group mean.
2 The absolute value of difference in head circumference from group mean (which disregards whether the difference is positive or negative).

Head circumference values were converted to z-scores on the basis of WHO references curves (World Health Organization, 2007). For infants who had data at two or more age points, a change in z-score was computed. We then used Pearson correlation to test for an association between individual-level peak node offset and:-

1 Change in z-score.
2 The absolute value of change in z-score (which disregards whether the change is positive or negative).

### 2.15 Combinatorial analysis

Subject parameter reconstruction represents the best-practice analysis given the available data on head size and array position for this dataset, while we consider constant parameter reconstruction to be an imaging equivalent to channel-space analysis. We conducted a combinatorial analysis to investigate the effect of group size on how the interpretations of subject parameter and constant parameter reconstructions may differ.

For each age and each hemisphere, a combinatorial analysis was conducted. To begin with, 100 sub-cohorts of 10 randomly chosen infants were selected. The same statistical analysis as was performed on the full cohort was performed on each sub-cohort of 10 randomly-selected infants. The position and cortical label of the peak node was determined for both the subject parameter and constant parameter reconstructions for each of the 100 sub-cohorts of group size 10, and used to calculate:

1 Mean peak node offset relative to subject parameter reconstructions for the group size.
2 The proportion of mismatching cortical labels for the group size.

This process was repeated for sub-cohort group sizes from 11 to the full cohort size.

## 3 Results

Of the datasets initially included in this study (N_5-months_ = 104, N_8-months_ = 97, N_12-months_ = 97), infants were excluded due to the following criteria:

• The infant was not tested
  ○ The infant was withdrawn (N_5-months_ = 3, N_8-months_ = 8, N_12-months_ = 9)
  ○ The infant missed the visit (N_5-months_ = 1, N_8-months_ = 2, N_12-months_ = 2)
• NIRS data acquisition was not undertaken at visit (N_5-months_ = 7, N_8-months_ = 9, N_12-months_ = 11)
• Task was not undertaken (no infants were excluded due to this criteria)
• The infant became fussy (N_5-months_ = 6, N_8-months_ = 5, N_12-months_ = 9)
• Experimental errors
  ○ PPhotographs of headgear placement missing (N_5-months_ = 5, N_8-months_ = 4, N_12-months_ = 1)
  ○ Video of infant during fNIRS data acquisition was missing (N_5-months_ = 8, N_8-months_ = 5, N_12-months_ = 3)
  ○ Missing event markers in the task (N_5-months_ = 1, N_8-months_ = 0, N_12-months_ = 0)
  ○ Other technical issues: data not saved due to a technical glitch or due to human error (N_5-months_ = 1, N_8-months_ = 1, N_12-months_ = 2)
• Poor placement of headgear (N_5-months_ = 5, N_8-months_ = 12, N_12-months_ = 6)
• Number of channels surviving channel pruning below minimum threshold (N_5-months_ = 5, N_8-months_ = 1, N_12-months_ = 1)
• Not enough valid trials for the auditory vocal condition (N_5-months_ = 3, N_8-months_ = 3, N_12-months_ = 4)
• Missing head circumference measurement (N_5-months_ = 6, N_8-months_ = 7, N_12-months_ = 4)

In total, our final sample size consisted of 53 infants aged 5-months (27 female, mean age±SD = 163.17 ± 12.15 days), 40 infants aged 8-months (19 female, 245.53 ± 8.36 days), and 45 infants aged 12-months (24 female, 376.16 ± 16.34 days).

### 3.1 Directly comparing image reconstruction to channel-space

**Fig. 5:**
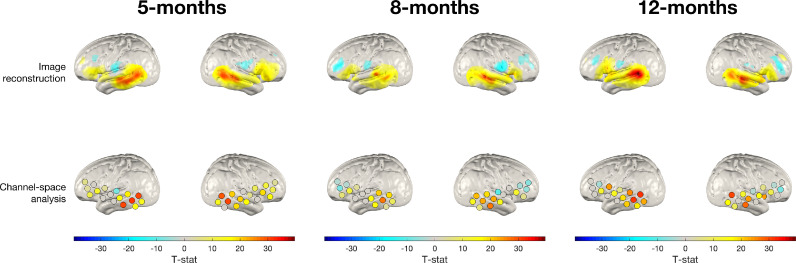
Group-level T-statistic images of changes in oxy-haemoglobin concentration in response to the auditory vocal condition relative to baseline for two approaches to analysing fNIRS data. Top row: subject parameter reconstruction pipeline. Bottom row: channel-space analysis. The significance level of displayed T-statistic values is *p* < 0.05, Bonferroni corrected on the basis of number of nodes in the grey matter surface mesh.

Firstly, the pipeline that represents the best-available practice image reconstruction, subject parameter reconstruction, and the channel-space analysis pipeline were compared. Fig. 5 shows group-level cortical T-statistic maps for changes in oxy-haemoglobin concentration in a 12–16 s post-stimulus window with respect to baseline for subject parameter reconstructions. Also shown in Fig. 5 are the cortical projections of group-level channel-wise T-statistic values comparing the same time windows.

The results appear very consistent between the two processing pipelines across all hemispheres at all ages in terms of spatial distribution, which is particularly true in the temporal lobe. In addition, at all age groups and across both hemispheres, the peak node and peak channel projection was observed in the middle temporal gyrus, demonstrating consistency in the results across the two processing streams at the group-level.

However, there are some areas where inferences differ, particularly in the inferior frontal regions, as can be seen in Fig. 5. In the left hemisphere at 5-months and the right hemisphere at 8-months, the group-level reconstructed images suggest larger changes in inferior frontal regions than can be inferred from channel-space, while in the right hemisphere at 12-months the group-level reconstructed image suggests more widespread concentration changes.

**Fig. 6:**
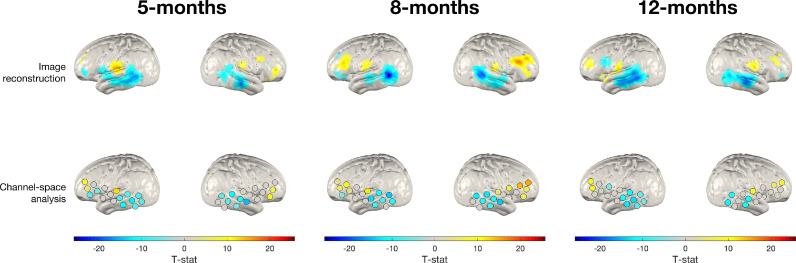
Group-level T-statistic images of changes in deoxy-haemoglobin concentration in response to the auditory vocal condition relative to baseline for two approaches to analysing fNIRS data. Top row: subject parameter reconstruction pipeline. Bottom row: channel-space analysis. The significance level of displayed T-statistic values is *p <* 0.05, Bonferroni corrected on the basis of number of nodes in the grey matter surface mesh.

Fig. 6 shows the same analysis repeated using the deoxy-haemoglobin signal, replicating the analysis seen in Fig. 5. As can be seen in Fig. 6, in general these results also appear broadly consistent with one another. The spatial distribution of activation suggested by the channel-space deoxy-haemoglobin T-statistic values appears to emulate what is seen in image-space. However, the T-statistic values themselves appear to be much lower lower in channel-space, which is particularly evident in the left hemisphere at all ages and the right hemisphere at 12-months.

In the deoxy-haemoglobin analysis, at all age groups and across both hemispheres, the peak node and peak channel projection was observed in the middle temporal gyrus, as was the case in the oxy-haemoglobin analysis.

**Fig. 7:**
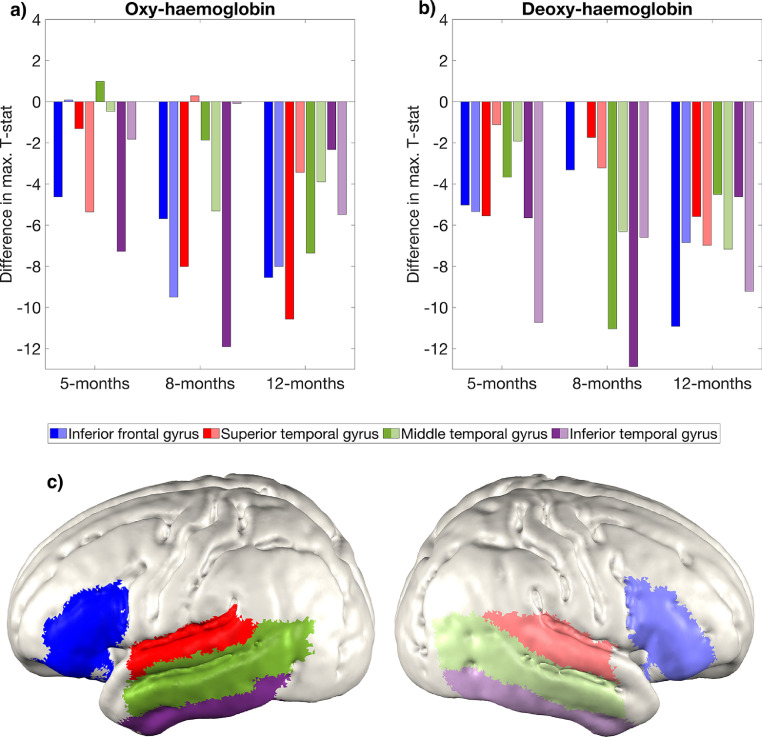
Differences in absolute maximum T-statistic values of the channel-space analysis relative to the subject parameter reconstruction for (a) oxy-haemoglobin and (b) deoxy-haemoglobin concentration changes across four cortical areas where activation is consistently seen in the oxy-haemoglobin channel-space analysis. For each pair of bars grouped by colour, the left bar represents the difference in that region in the left hemisphere and the right bar (with a more faded colour) represents the difference in that region in the right hemisphere. Note: at 8-months in the right hemisphere, no activation was seen in the inferior frontal gyrus in either the channel-space analysis or subject parameter group-level image. The cortical areas are shown in (c).

The cortical projections of channel-space positions showed that there were four cortical areas where significant changes in oxy-haemoglobin concentration occurred at the group-level: inferior frontal gyrus, superior temporal gyrus, middle temporal gyrus and inferior temporal gyrus. The maximum absolute T-statistic value of a channel projected to each of these four areas in each hemisphere was noted for both the oxy- and the deoxy-haemoglobin analysis. The maximum absolute T-statistic value was also taken for each of these four cortical areas in the subject parameter reconstruction. In Fig. 7, we show the difference between the channel-space maximum values and the subject parameter maximum values. Consistently, we see that channel-space underestimates the effect size relative to subject parameter reconstruction, and this is most evident at 12-months in the oxy-haemoglobin analysis and across ages in the deoxy-haemoglobin analysis.

### 3.2 Effects of variability in head size and array position

We also aimed to explore the effect that variability in head size and array position has on the analysis of fNIRS data. Comparisons were made at the group- and the individual-level between all of the four image reconstruction processing streams.

#### 3.2.1 Group-level

**Fig. 8:**
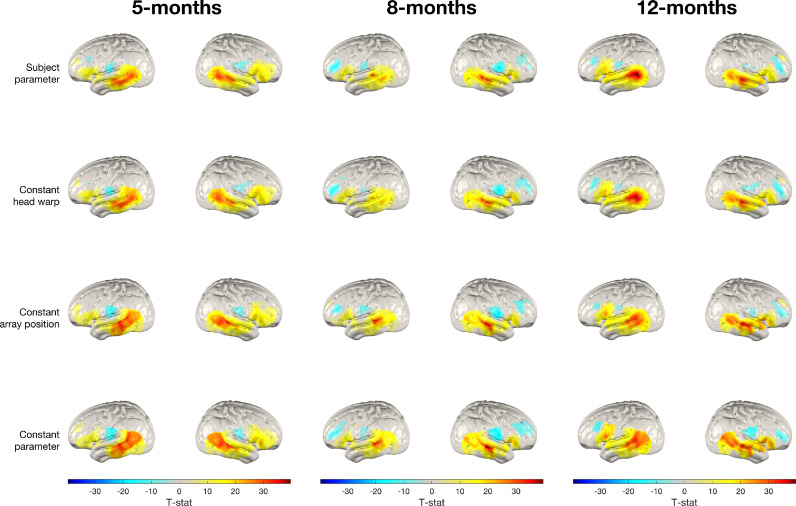
Group-level T-statistic images of changes in oxy-haemoglobin concentration in response to the auditory vocal condition relative to baseline for the four processing streams. From top row to bottom row: subject parameter reconstruction, constant head warp reconstruction, constant array position reconstruction, constant parameter reconstruction. The significance level of displayed T-statistic values is *p <* 0.05, Bonferroni corrected on the basis of number of nodes in the grey matter surface mesh.

Fig. 8 shows group-level oxy-haemoglobin T-statistic maps for the four different image reconstruction processing streams: subject parameter, constant head warp, constant array position and constant parameter. Qualitatively, it can be seen that the subject parameter and constant head warp reconstructions appear similar, suggesting that within-cohort variability in head size does not have a large impact on the resulting group level images. It can also be observed that the group-level constant parameter reconstruction images are similar to the group-level constant array position reconstruction images.

This qualitative observation is supported by the overlap between the images from the different processing streams where each group-level image is thresholded at 50% of its maximum value. Here, in both hemispheres at every age, we see greater levels of overlap between the thresholded constant head warp and subject parameter images than the other two processing streams (see Table 1). In each case, the 50% thresholded overlap is greatest between subject parameter images and constant head size images, though this is lowest (but still true) for the left hemisphere at 8-months. In this case, we see a less focal response across reconstruction pipelines, which is potentially due to this cohort having the smallest group size.

**Table 1:** Jaccard index quantifying overlap of the thresholded group-level image for each processing stream with the thresholded group-level subject parameter image. Group-level images from each processing stream were thresholded at 50% of their maximum value. A Jaccard index of 100% would indicate perfect overlap, while 0% would indicate no overlap at all.

Age	Number of infants	Group-level thresholded nodal overlap with subject parameter images (Jaccard index)					
		Left hemisphere			Right hemisphere		
		Constant head warp	Constant array position	Constant parameter	Constant head warp	Constant array position	Constant parameter
5-months	53	90.3%	71.9%	69.9%	83.0%	62.0%	58.2%
8-months	40	57.7%	48.1%	46.9%	74.2%	48.4%	50.6%
12-months	45	80.0%	61.9%	39.3%	78.8%	47.5%	39.7%

Despite the constant head warp pipeline appearing to most closely emulate the subject parameter pipeline, the group-level images across reconstruction pipelines are broadly consistent with one another. This suggests that group-level analyses are robust to variability in head size and array position.

**Fig. 9:**
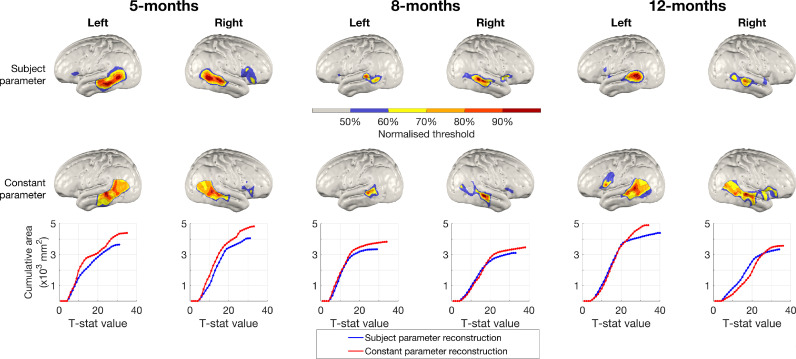
Top: normalised and thresholded group-level T-statistic images of changes in oxy-haemoglobin concentration in response to the auditory vocal condition relative to baseline for subject parameter (top row) and constant parameter (middle row) pipelines. Each image is thresholded at values between 50% and 90% of its maximum T-statistic value. Bottom row: cumulative area of activation as a function of T-statistic value. At larger T-statistic values, the area covered in subject parameter group-level images is consistently lower than is the case in the constant parameter group-level images, suggesting greater focality in images resulting from the subject parameter pipeline.

To compare focality and the spatial characteristics of activation between the subject parameter and constant parameter pipelines, each group-level T-statistic images from both pipelines for each age was normalised to its maximum value, and thresholded at values between 50% and 90% of that maximum value. This is shown in Fig. 9. On visual inspection, the subject parameter images appear more focal, while the spatial distribution of T-statistic values in the constant parameter group-level images appears more dispersed.

To better quantify this measure, we plot the cumulative area of activation as a function of T-statistic value in Fig. 9. Toward the maximum T-statistic values, the area covered in subject parameter group-level images is consistently lower than is the case in the constant parameter group-level images. This demonstrates greater focality in the subject parameter images.

#### 3.2.2 Individual-level

**Fig. 10:**
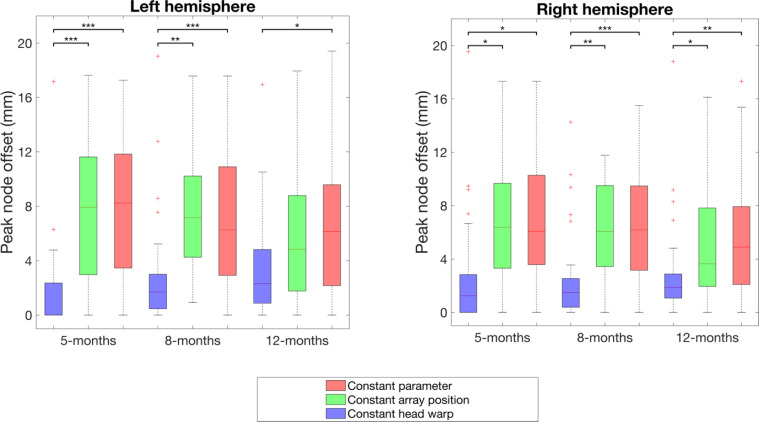
Peak node offset at the individual-level for each processing stream relative to subject parameter reconstructions. Peak node offset values were calculated in the space of the constant head warp model for each age. Significance levels were computed using paired t-tests. * represents *p <* 0.05 (corrected), ** represents *p <* 0.01 (corrected), *** represents *p <* 0.001 (corrected).

Fig. 10 shows the peak node offset at the individual-level for the three processing streams relative to subject parameter reconstructions. This analysis was conducted using images of oxy-haemoglobin concentration changes. It can be observed that the constant head warp peak node offset is substantially lower at each age in each hemisphere than the other two processing streams. The difference between individual-level peak node offset in the constant head warp and constant parameter pipelines is statistically significant in all cases, and this is also true for the difference seen between constant head warp and constant array position pipelines in all cases except the left hemisphere at 12-months.

We also quantified individual-level peak node offset between the subject parameter pipeline and the constant parameter pipeline modified such that the size of the head model at each age was determined by the average head measurements at the other two age points. This was performed to assess the effect of using a single-sized head model across all age cohorts. No statistically significant difference was found between peak node offset obtained using group-level age-matched head measurements and using non-age-matched measurements. That is to say, using non-age-matched warped head models does not perform noticeably worse than the constant parameter pipeline. This result is shown in *Supplementary Material*.

### 3.3 Peak node offset association with head size and growth trajectory

Though we see that array position is the dominant driver of differences between the subject parameter and constant parameter group-level images, we aimed to investigate whether there was any association between individual-level peak node offset and the magnitude of difference in head size from the group mean.

There were 24 individuals with data at both 5- and 8-months, 20 with data at both 8- and 12-months, and 25 with data at both 5- and 12-months, enabling an analysis by trajectory of head size. No correlation was observed between individual-level peak node offset and change in z-score or difference in head circumference from group mean at any age or for any of these age ranges. Further, for each comparison performed, no statistically significant correlations were found. As such, we have found no evidence to suggest inferences made from constant parameter reconstructions are systematically biased by either head size deviation from group mean or head growth trajectory. The full results for each comparison can be found in *Supplementary Material*.

### 3.4 Effect of group size

**Fig. 11:**
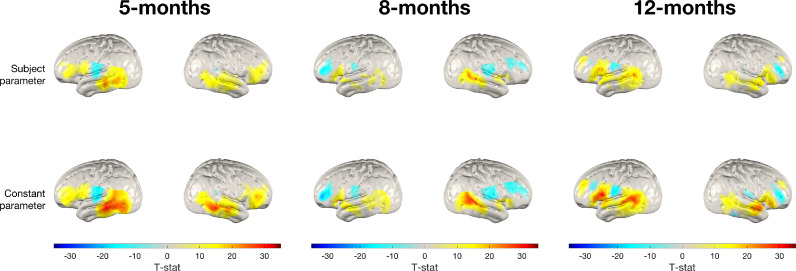
Group-level T-statistic images of changes in oxy-haemoglobin concentration in response to the auditory vocal condition relative to baseline for a sub-cohort of 10 randomly chosen infants at each age. Top row: subject parameter reconstruction pipeline. Bottom row: constant parameter reconstruction pipeline. The significance level of displayed T-statistic values is *p <* 0.05, Bonferroni corrected on the basis of number of nodes in the grey matter surface mesh.

In Fig. 11, we show group-level T-statistic response maps for a sub-cohort of 10 randomly selected infants at ages 5-, 8- and 12-months. On visual inspection, differences between the two processing streams at equivalent ages and for equivalent hemispheres are more apparent than was the case for the full-sized cohort in Fig. 8.

**Fig. 12:**
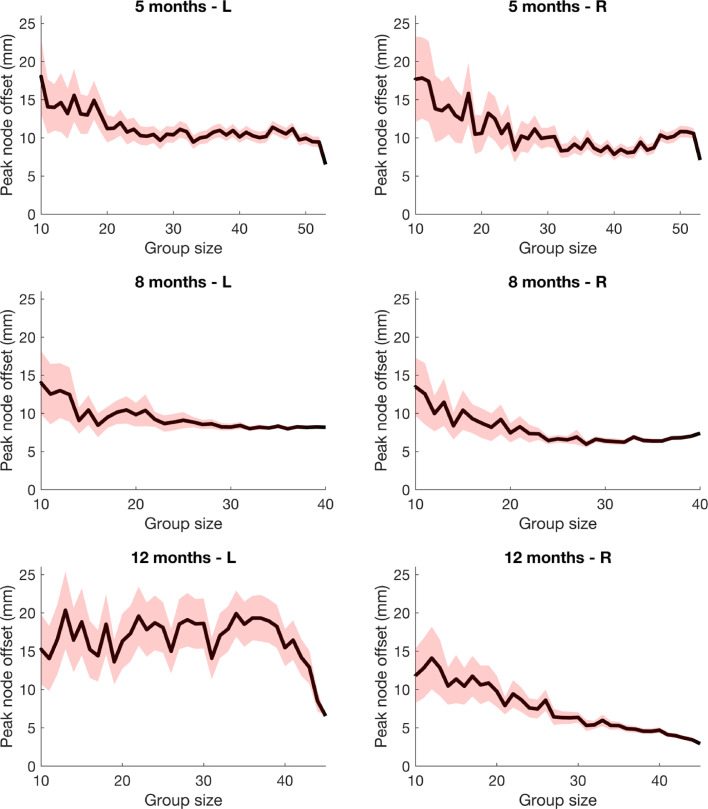
Peak node offset as a function of group size. Mean ± standard error is shown by the red shaded area. An increase in group size leads to a decrease in peak node offset and, by extension, a decrease in the likelihood of different inferences being drawn from the results at the group-level. Note: this effect is less evident at 12-months in the left hemisphere, but this likely relates to the fact that the constant parameter approach appears to yield two disparate peaks (one in the temporal lobe and one in the inferior frontal gyrus, see Fig. 8).

In Fig. 12, we plot mean peak node offset from groups of randomly assorted participants as a function of group size. In this context, we define peak node offset as the Euclidean offset between the position of the peak node in the group-level T-statistic images from subject parameter and constant parameter pipelines for each randomly assembled group. As group size increases, in general there is a decrease in the mean peak node offset.

**Fig. 13:**
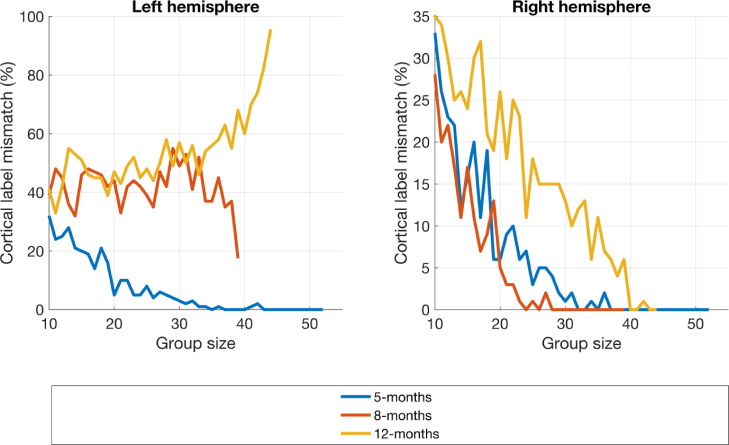
Cortical label mismatch between subject parameter and constant parameter reconstruction pipelines as a function of group size.

There is also decrease in the proportion of mismatched peak node cortical labels between the two processing streams as group size increases (see Fig. 13), except for the left hemisphere at 12-months.

## 4 Discussion

We have demonstrated an image reconstruction approach using fNIRS data acquired from a cohort of Gambian infants. Using image reconstruction to quantify and isolate the effects of variability in head size and array position, we find that inferences drawn from group-level channel-space fNIRS analyses are unlikely to be significantly affected by these assumptions given the variability of these parameters in our dataset, though their effect is much more influential at the individual-level. We find that variability in array position is the dominant factor that drives differences between channel-space analysis and best-practice image reconstruction at the individual- and group-level. Our combinatorial analysis shows that the influence of variability in array position and head size on statistical and anatomical inferences is weakened as group size increases.

### 4.1 Inferences from channel-space and image reconstruction

This analysis sought to directly compare group-level analyses of our data in channel-space to subject parameter reconstruction, which represents a best-practice image reconstruction pipeline given the available data on head size and array position. As was shown in Figs. 5 and 6, group-level subject parameter reconstructions and channel space projections are notably consistent across the two processing streams. For both the oxy- and deoxy-haemoglobin analysis at the group-level, areas where we see activation in the channel space projections are also where we see activation in subject parameter reconstructions, especially in the temporal lobe, demonstrating consistency between both processing streams at the group-level. In addition, the cortical labels of the peak node and peak channel were consistent between the two processing streams for each age, hemisphere and chromophore, which further demonstrates consistency. This direct qualitative comparison of channel-space and best-practice image reconstruction included in this analysis provides evidence that the influence of variability in head size and array position on statistical and anatomical inferences is weakened at the group-level.

For large effect sizes and large group sizes, it is unlikely that analysing data in channel-space will lead to substantially different inferences about activation; however, for smaller effect sizes, an analysis using subject parameter image reconstruction will likely better resolve the effect. In our data, the channel-space analysis consistently underestimates the effect size seen in image space (see Fig. 7). This likely pertains to the fact that image reconstruction uses models of light transport to account for differences in channel sensitivity across subjects, and thus when an average is taken, it is more likely that the signals contributing to that average are derived from the same cortical regions across subjects. Though we are using functional data where a large effect size is expected, the fact that image reconstruction still results in greater significance suggests that investigations of smaller differences (for example, age-related differences within a longitudinal cohort, investigations of the deoxy-haemoglobin response and studies with smaller group sizes) may benefit from an image-based analysis.

### 4.2 Effect of head size and array position

The analyses presented aimed to isolate the effects of variability in head size and array position seen in our dataset on the analysis of fNIRS data. The constant parameter reconstruction pipeline represents an imaging approach that is conceptually equivalent to a channel-space analysis, insofar as it assumes a fixed array that is consistent across participants, as well as assuming a fixed model of the cerebral anatomy. In addition, the effect of variability in head size and array position can be isolated using subject-specific values for one parameter and group-average values for the other.

At the individual-level, our results show that differences between subject parameter and constant parameter reconstructions are driven by variability in array position. It can be seen in Fig. 10 that constant head warp reconstruction leads to the lowest level of peak node offset, while not accounting for array position (whether or not accounting for head size) leads to a much greater degree of peak node offset. In addition, we found no statistically significant differences between individual-level peak node offset obtained using group-level age-matched head measurements and using non-age-matched measurements (see *Supplementary Material*). Our results show that using any single head model for all infants (warped to a size within the 5-12 month range) does not result in significantly worse performance than our constant parameter pipeline that uses group-level age-matched warping. This conclusion is anticipated given the dominance of variability in array position in driving different inferences.

The images obtained via constant head warp and subject parameter approaches at the individual- and the group-level are notably more similar than the other two pipelines are in comparison to subject parameter (see Figs. 8 and 10). Our results show that the difference between the subject parameter and constant parameter pipelines is primarily driven by variability in array position and not in head size. We therefore conclude that collecting data on array positioning from each infant and employing an image reconstruction approach is essential to increase the reliability of fNIRS data analysis at the individual-level. Subject parameter reconstruction produces images with greater focality (see Fig. 9); as such, accounting for subject-specific parameters can increase confidence in the spatial localisation of activation, and is likely able to better resolve features of activation particularly for smaller group sizes than is the case in channel-space analysis or constant parameter reconstruction.

One of the biggest differences between subject parameter and constant parameter images at the group-level is in the inferior frontal gyrus; this is particularly evident at 12-months in the left hemisphere where we see a larger peak T-statistic value in the inferior frontal gyrus in the constant parameter image. The differences seen in frontal regions may well be due to their slightly greater depth underneath the scalp, and so size and shape of the head model will have a greater impact on modelling photon transport in frontal areas than is the case for shallower cortical regions. Brain activation at a greater depth will influence fewer measured photons than activation at a shallower depth, and so activation occurring deeper will have a reduced signal-to-noise ratio.

We sought to investigate whether within-cohort head size variation and growth trajectory were associated with peak node offset in constant parameter reconstructions, a surrogate of whether there is a systematic bias in channel-space analysis linked to these factors. We found no evidence that such a link exists; the correlation between head circumference deviation from group mean or change in z-score and peak node offset was not significant for any correlation tested. In addition, there is also no clear pattern of peak node offset being larger at the extremes of either of these metrics (see *Supplementary Material*). This signifies that there is no systematic error linked to these factors in channel-space analysis.

Fundamentally, combining data from equivalent channels across individuals implicitly assumes that equivalent channels probe equivalent anatomical volumes of the cortex. One possible explanation for why variability in array position has a substantially larger influence than variability in head size is due to correspondences between scalp positions and underlying anatomy. Tsuzuki et al. (2017) demonstrated using a cortical projection method that the 10-10 system is sufficient to predict underlying macroanatomical cortical structures in infants from birth to 2 years. This suggests that an array positioned on two different sized heads (within a plausible anatomical range for a given age) is likely to be overlying the same cortical area. In contrast, if an array position deviates from the group-average position, it is very likely to be overlying (and, by implication, sampling) different regions of the cortex to what the average array position would suggest.

The effect of variability in array position appears to have a substantial influence on peak node offset at the individual-level but its influence is diminished at the group level. This exhibits the robustness of assuming constant head size and array position (a fundamental assumption implied in channel-space analysis) at the group-level in fNIRS analyses. This finding is consistent with findings reported by Blasi et al. (2014), who found high test-retest reliability of group-level oxy-haemoglobin response in infants aged 4- to 12-months, but much lower reliability at the individual-level.

### 4.3 Combinatorial analysis and the effect of group size

In the combinatorial analysis, for each age and hemisphere, peak node offset decreases as group size increases; in other words, the constant parameter results converge towards (but never meet) the subject-parameter results as group size increases. We conclude that this decrease shows that the effect of the variability in head size and array position becomes less evident as more individuals are included in each group. This has significant implications for channel-space analyses, where head size and array position are also generally assumed constant. Subject parameter reconstruction approaches are likely the superior analysis approach, but their benefit is particularly evident for smaller group sizes.

In all cases except for the left hemisphere at 12-months, the proportion of mismatched cortical labels between the two processing streams decreases as group size increases. At 12-months in the left hemisphere, there appears to be a broad focus that straddles the superior and middle temporal gyri, which helps to explain how a mismatch in peak node cortical label could have occurred at the full cohort size.

Our results demonstrate that there is a weakening of the effect of variability in head size and array position as group size increases. Though our results suggest that assuming these parameters constant is questionable at the individual-level, the influence of variability in these parameters (to the extent seen in our dataset) is weakened as group size increases. This further supports our claim that channel-space fNIRS analyses are robust to variability in these parameters in longitudinal infant studies at the group-level. Our analysis does not allow us to address the broader question as to what number of participants is an appropriate cohort size for field-based fNIRS studies; this question is highly dependent on the expected size of activation and other parameters of the experiment in question.

As we have been using infant-specific data, we cannot state whether similar results would be observed across different cohorts (e.g. adults with varying head sizes). However, given that the fundamental characteristics of the problem (optodes manually coupled to a head) are consistent across all ages, it does seem likely that an increasing group size will increase the robustness of the channel-space analysis, and that this robustness would be further increased (particularly when the cohort size is small) when a subject parameter image reconstruction approach is employed. In addition, this conclusion should be independent of the statistical analysis used; for example, a generalised linear model analysis should also benefit from employing subject parameter reconstruction.

### 4.4 The benefit of an image reconstruction approach

Image reconstruction techniques are better suited to high-density arrays that contain overlapping channels (Boas et al., 2004; White, 2010), particularly if they also include a range of source-detector separations, which allow depth discrimination: this form of image reconstruction is known as diffuse optical tomography (DOT) (Lee et al., 2017). A recent example of high-density DOT being applied in a field-based context is a study by Fishell et al. (2020) of Colombian children. A notable example of a high-density DOT study in infants was conducted by Frijia et al. (2020) using the same paradigm as was used in this present study.

An image reconstruction approach produces images inherently registered to the head model, allowing concentration changes to be visualised on a model of cortical anatomy (Yücel et al., 2017). Parcellation atlases can be incorporated into the analysis of reconstructed images in a similar fashion to how the AAL atlas was used in in this work, permitting cortical labels to be attributed to nodes or voxels in the head model which enables the response in equivalent cortical areas to be compared across populations.

In addition, using anatomical information present in the head model, reconstructed images can be registered to a common space to be compared with data acquired from several complimentary functional imaging modalities such as electroencephalography (EEG), magnetoencephalography (MEG), and fMRI. This can enable longitudinally-acquired fNIRS data to be compared directly to fMRI data collected from child and adult populations, helping to bridge gaps in our understanding of functional development. Our work represents a significant step towards enabling such comparisons for longitudinal infant populations, particularly those in field-based studies. Such comparisons between fMRI and fNIRS data collected with high-density arrays have been conducted in adults (for example, (Eggebrecht et al., 2014)).

Image reconstruction incorporates models of light transport in the analysis of fNIRS data (Arridge and Cooper, 2015). Models of light transport have also been employed to infer a cortical label of activation without taking an image reconstruction approach, such as was performed by Perdue et al. (2019). However, such an approach still confines statistical inferences about functional activation to a discretised and arbitrary channel-space.

The reconstruction of spatially-continuous images of concentration changes on the cortex removes the need to assume a given channel and scalp location is associated with a single cortical position. Constraining analysis to a discretised channel-space does not enable the intricacies of the spatial characteristics of activation on the cortex itself to be investigated. Image reconstruction approaches can enable the development of longitudinal changes in the spatial distribution and focality of functional responses to be studied, which we have begun to investigate in this work.

To our knowledge, Wijeakumar et al. conducted the only previous longitudinal image reconstruction study in a field-based setting with infants in this age range that has been published (Wijeakumar et al., 2019), though this study does not explicitly investigate longitudinal imaging of infants aged 12-months and under, and has far fewer participants than the cohorts in our analysis. Our work therefore forms the foundation of field-based longitudinal image reconstruction in infants up to 12-months of age. Improvements in infant image reconstruction approaches, building on the demonstration in this work, can help improve the localisation error and resolution of infant image reconstruction, but must also focus on doing so in the context of a low-resource setting where acquiring subject-specific MRI data, digitised optode positioning data and using high-density arrays may not be feasible.

### 4.5 Head modelling

A model of head anatomy is required for image reconstruction. A head model would ideally be obtained from an individual's own MRI scan so as to be subject-specific; however, this approach was not feasible for the BRIGHT project. In the age range here, there are several sources of age-appropriate MRI data. In this work, we have used a head model built from structural data acquired and pre-processed by Shi et al. (2011) which we aim to include in our group's toolbox on Github (www.github.com/DOT-HUB). An MRI atlas for infants aged 6-months was constructed by Akiyama et al. (2013). For structural head models at several age points in the range in this work, there exists data published in the Neurodevelopmental MRI Database (Richards et al., 2016; Richards and Xie, 2015; Sanchez et al., 2012). Our group has also published several models that are available via our website (www.ucl.ac.uk/dot-hub).

In this work, we do not use age-specific head models. We conducted a preliminary sensitivity analysis on forward modelling using array position data from five arbitrarily chosen infants at 5-months, and used age-appropriate models (from the Neurodevelopmental MRI Database) to investigate the effect of longitudinal changes in anatomy as well as array position, head size, and cranial landmark positions on light transport. We found that the median variation in channel centre of mass as a result of longitudinal anatomical changes for each array position was 3.0 mm (median absolute deviation 1.1 mm), substantially smaller than the effect of variability in array position (median 7.4 mm, median absolute deviation 3.8 mm) and statistically significant (*p* < 0.0001). The full results are shown in *Supplementary Material*.

The level of influence of longitudinal anatomical changes on light transport must also be placed in the context of our array registration method. Qualitatively, it can be seen in Fig. 4 that our method is a reasonable approximation of array position, and the data used to register the array was extracted specifically from participants. However, given that we did not collect data using a digitised positioning system or employ sophisticated photogrammetry methods, we have not been able to conduct a quantitative assessment of our array registration method. The error in the sensitivity distribution resulting from the array registration process is likely to be larger than the effect of longitudinal anatomical changes.

Another benefit of using the same model across ages is that there is an explicit one-to-one nodal correspondence regardless of how the model has been warped. This removes the need to register different head models to a common space, which itself would be liable to a degree of error. This one-to-one nodal correspondence was used to display group-level images in a common space across ages, as is shown in Figs. 5, 6, 8, 9 and 11.

In addition, given that we employed a cortically-constrained reconstruction method, we desired a cortical surface clearly displaying the principal gyri and sulci, which was another factor affecting our choice of head model. A more well-defined cortical surface could be extracted using the Shi et al. averaged data, which was accompanied by the registered AAL atlas, than was the case with the Sanchez et al. averaged data.

No MRI data was available from a cohort of West African infants to build a head model. It is unclear how the head model in this work, derived from infants living in a high-income country, may bias our results when used to represent head structure of Gambian infants. This is impossible to ascertain given the current lack of structural MRI data from this population, which may be difficult to obtain given the lack of MRI units in West Africa, in particular high-field imaging systems (Ogbole et al., 2018). In addition, we had no access to any individual-level data from infants at the ages under investigation. This underlines the need for more publicly-available high-quality MRI data from infants in this age range, similar to the database of individual-level structural priors presented by Collins-Jones et al. (2020).

### 4.6 Future work

This study validates channel-space fNIRS inferences drawn at the group-level, and helps bolster confidence in conclusions drawn from previous fNIRS studies in longitudinal cohorts. However, as has been outlined, an image reconstruction approach to analysing fNIRS data in its own right can be beneficial. While image reconstruction analyses are still relatively rare in fNIRS, they are not excessively complex and are likely to become ubiquitous in the coming years. Several packages are now available that can undertake image reconstruction-based processing (e.g. Homer3 (https://openfnirs.org/software/homer/), NeuroDOT (Eggebrecht and Culver, 2019)). The tools we used in this work are part of the DOT-HUB toolbox and are already available open-source (www.github.com/DOT-HUB).

The image reconstruction pipeline demonstrated in this work incorporates models of light transport, enables anatomical and functional data to be related to one another, and enables the spatial characteristics of activation to be investigated and understood. As such, we envisage widespread use of image reconstruction in future publications of longitudinal infant fNIRS studies.

## 5 Conclusion

Using an image reconstruction approach to analyse longitudinally-acquired infant fNIRS data, we have found that inferences drawn from group-level channel-space fNIRS analyses are robust to the implicit assumptions of constant head size and array position. We found that variability in array position, not head size, is the dominant factor that drives differences between channel-space and image-space analyses at the group- and the individual-level. In addition, we have shown that the influence of array position variability diminishes as group size increases. We envisage that the use of image reconstruction in longitudinal infant fNIRS studies will become widespread to permit the incorporation of anatomical information in data analysis and has the potential to enable the combination of functional data across modalities.

## Data and code availability statement

The data analysed in this work were acquired as part the BRIGHT project, which is running under the ethical approval of the joint Gambia Government/MRC Unit The Gambia Ethics Committee. Requests for access to anonymised copies of the data collected in BRIGHT can be made to the study PI (c.elwell@ucl.ac.uk), and will be subject to further approval from the Ethics Committee in The Gambia. The code used in this paper has been developed and released via www.github.com/DOT-HUB.

## Author statement

**Liam H. Collins-Jones:** Conceptualisation, Software, Formal analysis, Investigation, Data Curation, Writing- Original Draft, Writing - Review and Editing, Visualisation.

**Robert J. Cooper:** Conceptualisation, Software, Investigation, Resources, Writing - Review and Editing, Supervision.

**Chiara Bulgarelli:** Formal analysis, Investigation, Data Curation, Writing - Review and Editing.

**Anna Blasi:** Conceptualisation, Investigation, Data Curation, Writing - Review and Editing.

**Laura Katus:** Investigation, Writing - Review and Editing.

**Samantha McCann:** Investigation, Data Curation, Project administration.

**Luke Mason:** Conceptualisation, Software.

**Ebrima Mbye:** Investigation.

**Ebou Touray:** Investigation, Data Curation.

**Mohammed Ceesay:** Investigation.

**Sophie E. Moore:** Writing - Review and Editing, Investigation, Project administration, Supervision, Funding acquisition.

**Sarah Lloyd-Fox:** Conceptualisation, Investigation, Writing - Review and Editing, Project administration, Supervision, Funding acquisition.

**Clare E. Elwell:** Investigation, Writing - Review and Editing, Project administration, Supervision, Funding acquisition.

**Lena Acolatse:** Investigation, Data Curation, Project administration.

**Maria Magdalena Crespo-Llado:** Data Curation.

**Momodou Darboe:** Supervision.

**Saikou Drammeh:** Investigation.

**Tijan Fadeira:** Investigation.

**Giulia Ghillia:** Data Curation.

**Buba Jobarteh:** Investigation.

**Marta Perapoch Amado:** Data Curation.

**Andrew Prentice:** Funding acquisition.

**Maria Rozhko:** Data Curation.

**Mariama Saidykhan:** Investigation.

## Declaration of Competing Interest

R.J.C. has financial interests in Gowerlabs Ltd, a manufacturer of fNIRS technologies.

## Appendix Supplementary materials

Image, application 1
