# Supplementary material for: Longitudinal infant fNIRS channel-space analyses are robust to variability parameters at the group-level: An image reconstruction investigation
Source: Neuroimage. 2021 Aug 15;237:118068. doi: 10.1016/j.neuroimage.2021.118068 (PMC8285580; doi:10.1016/j.neuroimage.2021.118068)
Supplement: Supplementary file 1 [file mmc1.docx]

**Supplementary material**

**S1. Preliminary sensitivity analysis**

We conducted a preliminary analysis to determine the influence of longitudinal changes in structural anatomy and other factors on light transport. We built head models from age-appropriate averaged MRI data presented in Sanchez et al. (2012) using a method similar to that which is described in *Methods*. Three head models were produced: one using data at 6-months, one using data at 9-months, and another using data at 12-months. The array positioning data at 5-months was chosen arbitrarily from 5 infants. We then conducted four different analyses of sensitivity.

**Native model space**

In the first analysis, array positioning data for each of the 5 chosen was used to register the array to the (unscaled and unwarped) 6-months, 9-months and 12-months head models. Light propagation was then simulated using Toast++ (as is described in *Methods*). For each model, the cortical position of the centre of mass of the sensitivity distribution for each channel was computed, using a weighted sum of node coordinates where each node’s sensitivity value was used as a weight.

Therefore, there were three centre of mass positions for each channel across all ages. For a given array position, all centre of mass positions were transformed to the space of the 6-month model using ANTS registration. For a given array position, the Euclidean separation between each equivalent channel position at each age in the common space was computed and combined across channels; we refer to this as *between-age variability* in channel position.

In addition, there were five centre of mass positions for each channel at a given age. For each of the three ages, the Euclidean separation between each equivalent channel position was computed and combined across channels; we refer to this as *within-age variability* in channel position.

The resulting variability will give us an idea of how variability in array position affects light transport. We further identified three main sources of potential variation whose effects we sought to isolate:-

1. Variation in cranial landmarks.
2. Variation in head size.
3. Longitudinal anatomical changes.

**Longitudinal anatomical changes**

To isolate the effect of longitudinal changes in anatomy on light propagation, the above process was repeated with two key differences:-

1. Each head model was scaled to be the same head circumference as the 6-month head model.
2. The scalp positions from the 6-month model were used to register the array to the head model.

**Variation in head size**

To isolate the effect of head size, the head model created using data from Shi et al. (2011). For each age, this head model and its cranial landmarks were scaled to be the same circumference as the age-appropriate head models created using Sanchez et al. data.

**Variation in cranial landmarks positions**

The positions of cranial landmarks were determined on the surface of each model created using the Sanchez et al. data, and so were determined independently. Using ANTS, the registration between the head mask from Sanchez et al. data and Shi et al. data was computed, and was used to transform the positions of the cranial landmarks from each age-appropriate model to the space of the (unscaled and unwarped) Shi et al. model.

**Results**

Supplementary figure 1 shows the between-age variability as well as the within-age variability in channel centre of mass for each of the four analyses described. The most interesting plot is the one concerning longitudinal anatomical changes (the second row of the figure). The median between-age variability is 3.0 mm, which is a measure of the effect of longitudinal changes in anatomy on light transport (given the available age-appropriate averaged structural data). However, the within-age variability, where centre of mass positions were computed using different array positions, is substantially greater. This indicates that the effect of longitudinal changes in anatomy on light transport is much lower than the influence of array position, and this information helped inform our decision to use the Shi et al. data to produce a single model used across ages in our work; this is further addressed in *Discussion*.


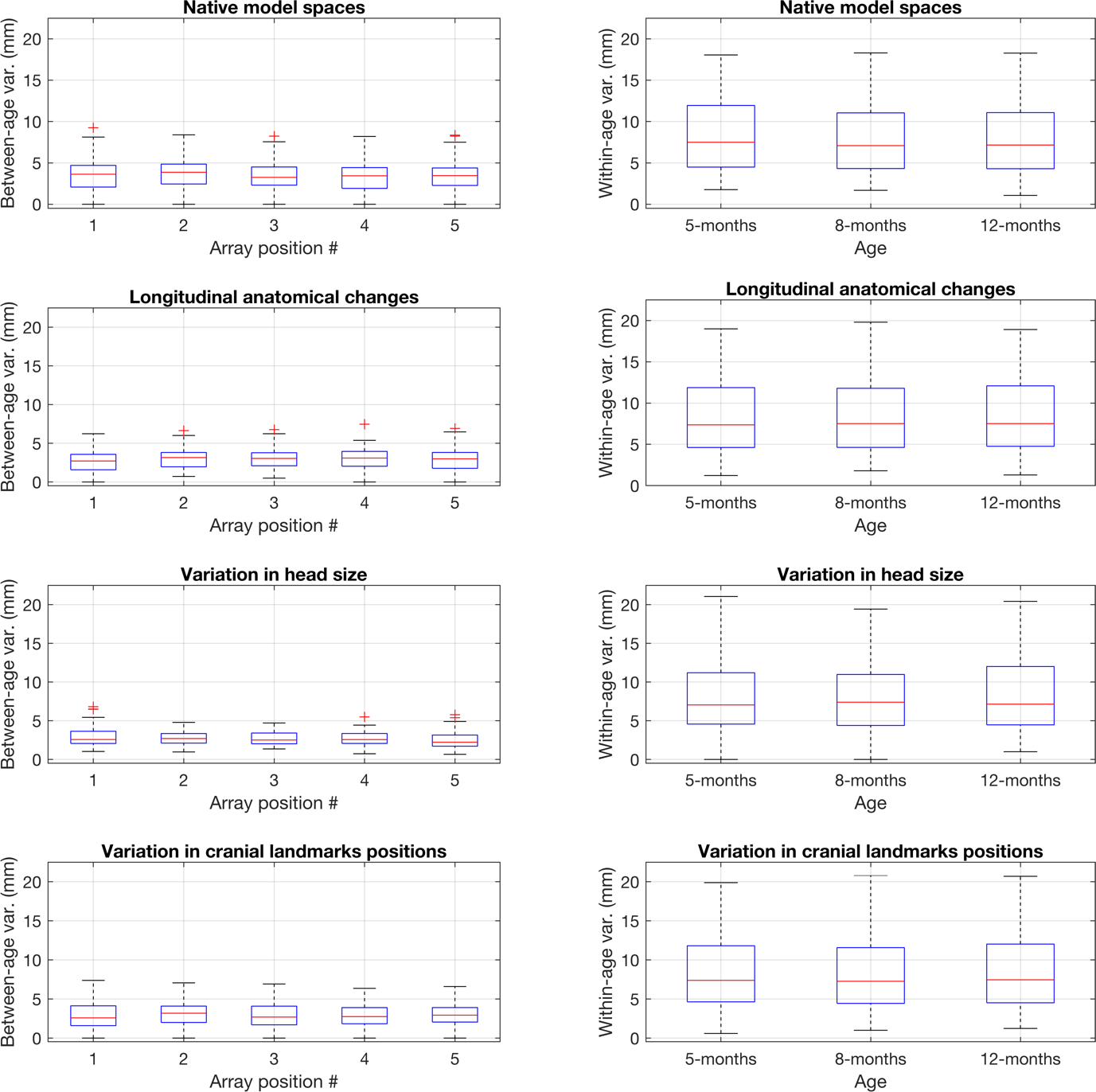


Supplementary figure 1: Between-age and within-age variability quantifying the effects of the different parameters investigated in the sensitivity analysis on light transport.

**S2. Relationship between peak node offset and longitudinal growth measures**

We investigated the relationship between head circumference, head growth trajectory and peak node offset. In total, we conducted 36 Pearson Correlation tests to test for an association between:-

- Peak node offset and difference in head circumference from group mean (1 test per age, per hemisphere, 6 in total). See results in Supplementary table 1.
- Peak node offset and absolute difference in head circumference from group mean (1 test per age, per hemisphere, 6 in total). See results in Supplementary table 2.
- Change in head circumference z-score and peak node offset (1 test per age, per age range, per hemisphere, 12 in total). See results in Supplementary table 3.
- Absolute value of change in head circumference z-score and peak node offset (1 test per age, per age range, per hemisphere, 12 in total). See results in Supplementary table 4.

Supplementary table 1: Peak node offset & difference in head circumference from group mean

| **Age** | **Hemisphere** | | | |
| --- | --- | --- | --- | --- |
|  | **L** | | **R** | |
| 5-months | r = 0.15 | p = 0.30 | r = 0.18 | p = 0.19 |
| 8-months | r = -0.12 | p = 0.48 | r = -0.01 | p = 0.97 |
| 12-months | r = 0.20 | p = 0.19 | r = -0.15 | p = 0.33 |

Supplementary table 2: Peak node offset & absolute difference in head circumference from group mean

| **Age** | **Hemisphere** | | | |
| --- | --- | --- | --- | --- |
|  | **L** | | **R** | |
| 5-months | r = 0.13 | p = 0.35 | r = -0.02 | p = 0.87 |
| 8-months | r = -0.13 | p = 0.43 | r = -0.29 | p = 0.07 |
| 12-months | r = 0.05 | p = 0.72 | r = 0.09 | p = 0.55 |

Supplementary table 3: Peak node offset & change in head circumference z-score

| **Age range** | **Age** | **Hemisphere** | | | |
| --- | --- | --- | --- | --- | --- |
|  |  | **L** | | **R** | |
| 5- to 8-months | 5-months | r = 0.05 | p = 0.83 | r = 0.13 | p = 0.54 |
|  | 8-months | r = 0.38 | p = 0.07 | r = 0.11 | p = 0.62 |
| 8- to 12-months | 8-months | r = 0.02 | p = 0.95 | r = 0.01 | p = 0.95 |
|  | 12-months | r = 0.38 | p = 0.10 | r = 0.26 | p = 0.27 |
| 5- to 12-months | 5-months | r = 0.10 | p = 0.65 | r = -0.05 | p = 0.81 |
|  | 12-months | r = 0.00 | p = 1.00 | r = 0.22 | p = 0.28 |

Supplementary table 4: Peak node offset & absolute change in head circumference z-score

| **Age range** | **Age** | **Hemisphere** | | | |
| --- | --- | --- | --- | --- | --- |
|  |  | **L** | | **R** | |
| 5- to 8-months | 5-months | r = -0.21 | p = 0.32 | r = -0.18 | p = 0.40 |
|  | 8-months | r = 0.08 | p = 0.70 | r = -0.09 | p = 0.66 |
| 8- to 12-months | 8-months | r = -0.12 | p = 0.63 | r = -0.11 | p = 0.65 |
|  | 12-months | r = -0.16 | p = 0.49 | r = -0.34 | p = 0.15 |
| 5- to 12-months | 5-months | r = -0.17 | p = 0.42 | r = 0.01 | p = 0.97 |
|  | 12-months | r = -0.04 | p = 0.84 | r = -0.07 | p = 0.74 |


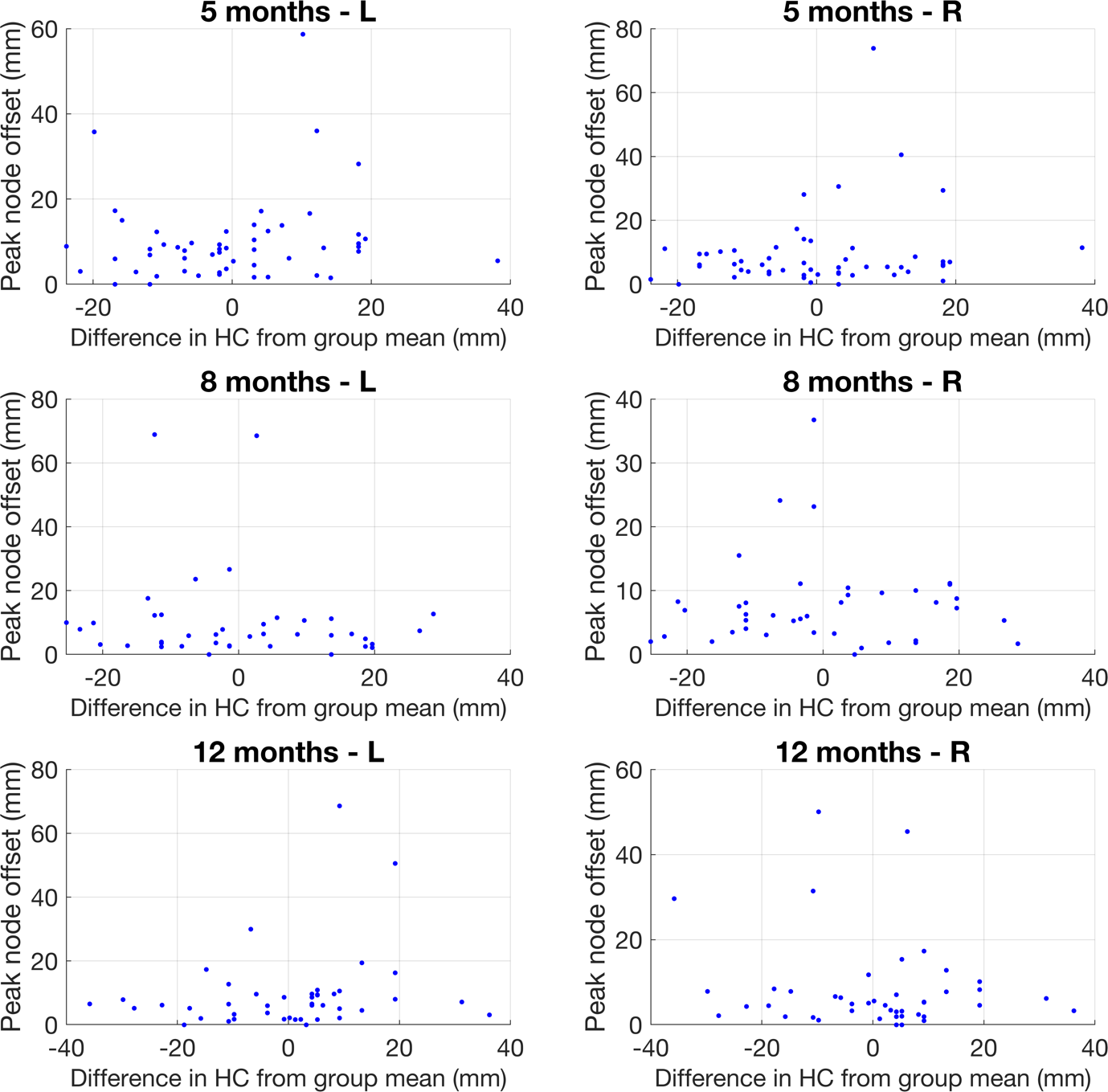


Supplementary figure 2: Peak node offset plotted against difference in head circumference from group mean for each age and hemisphere.


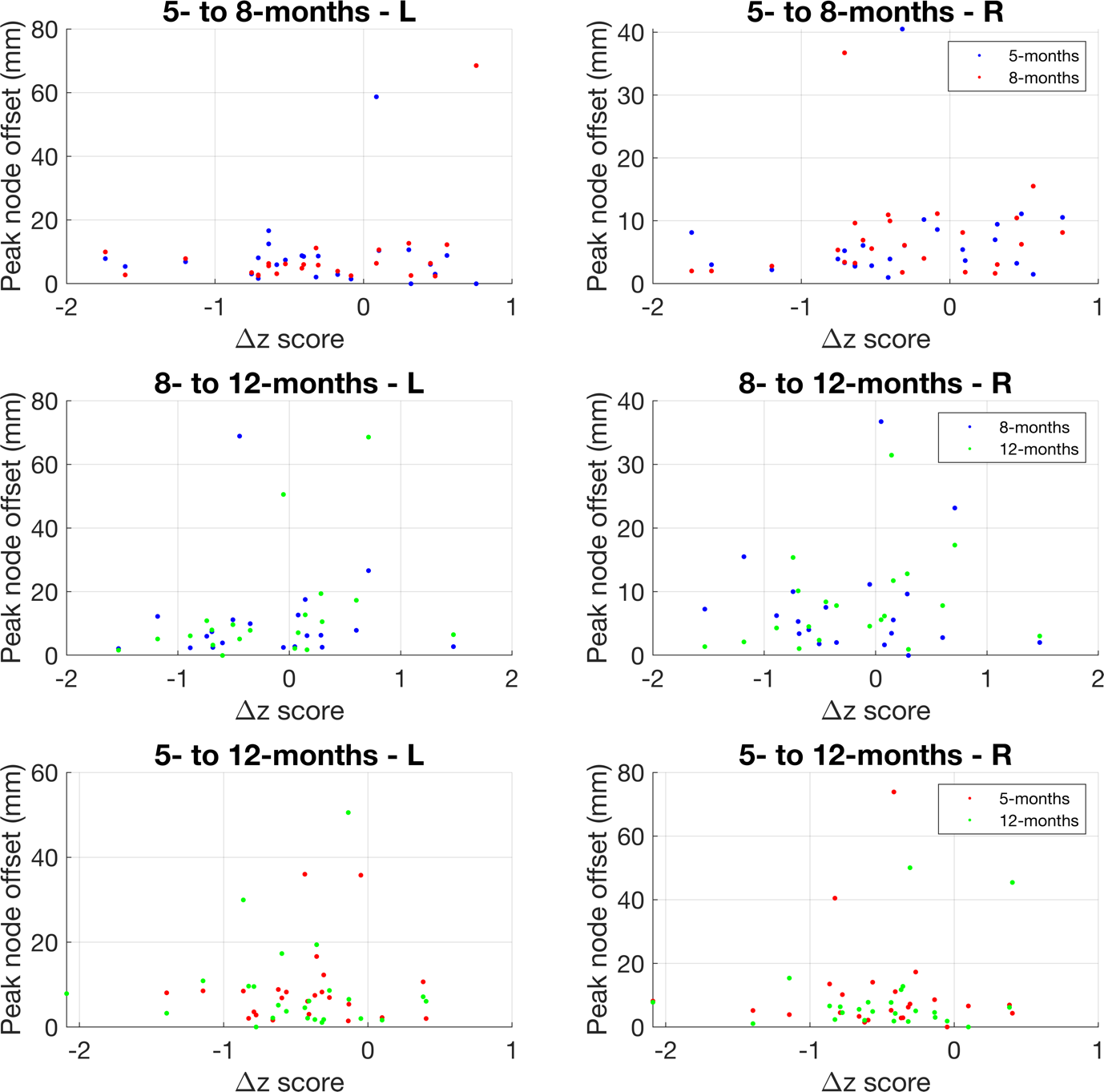


Supplementary figure 3: Peak node offset plotted against change in head circumference z-score between selected age points.

**S3. Accuracy of head warp**


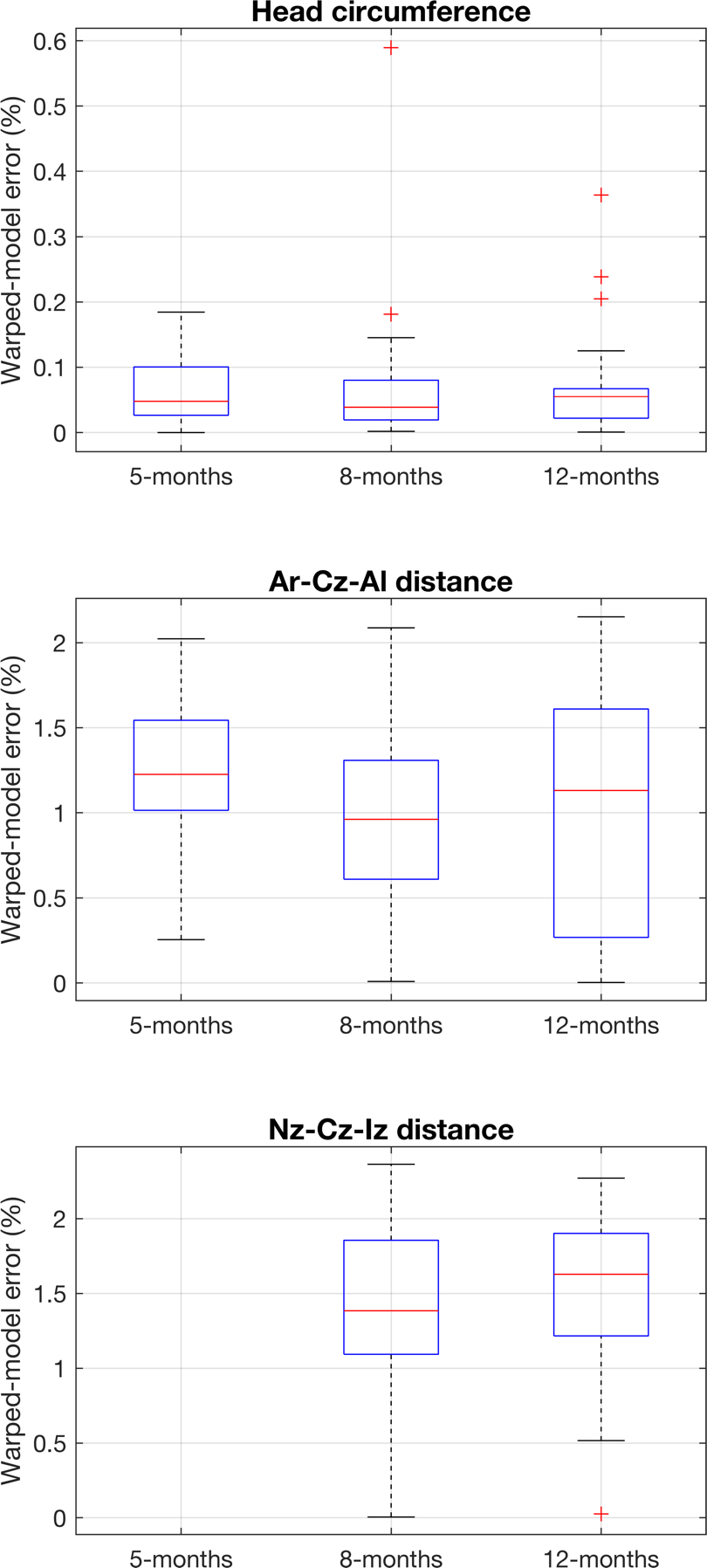


Supplementary figure 4: The error of head measurements from the warped head model in the subject parameter pipeline relative to the infant-measured values which were used as parameters to optimise the head warping process. Note: no Nz-Cz-Iz measurements were available for infants aged 5-months.

**S4. Graphical representation of paradigm**

Supplementary figure 5: Representation of stimulus presentation in the experimental paradigm.

**S5. Peak node offset using non-age-specific head measurements for warping**

**
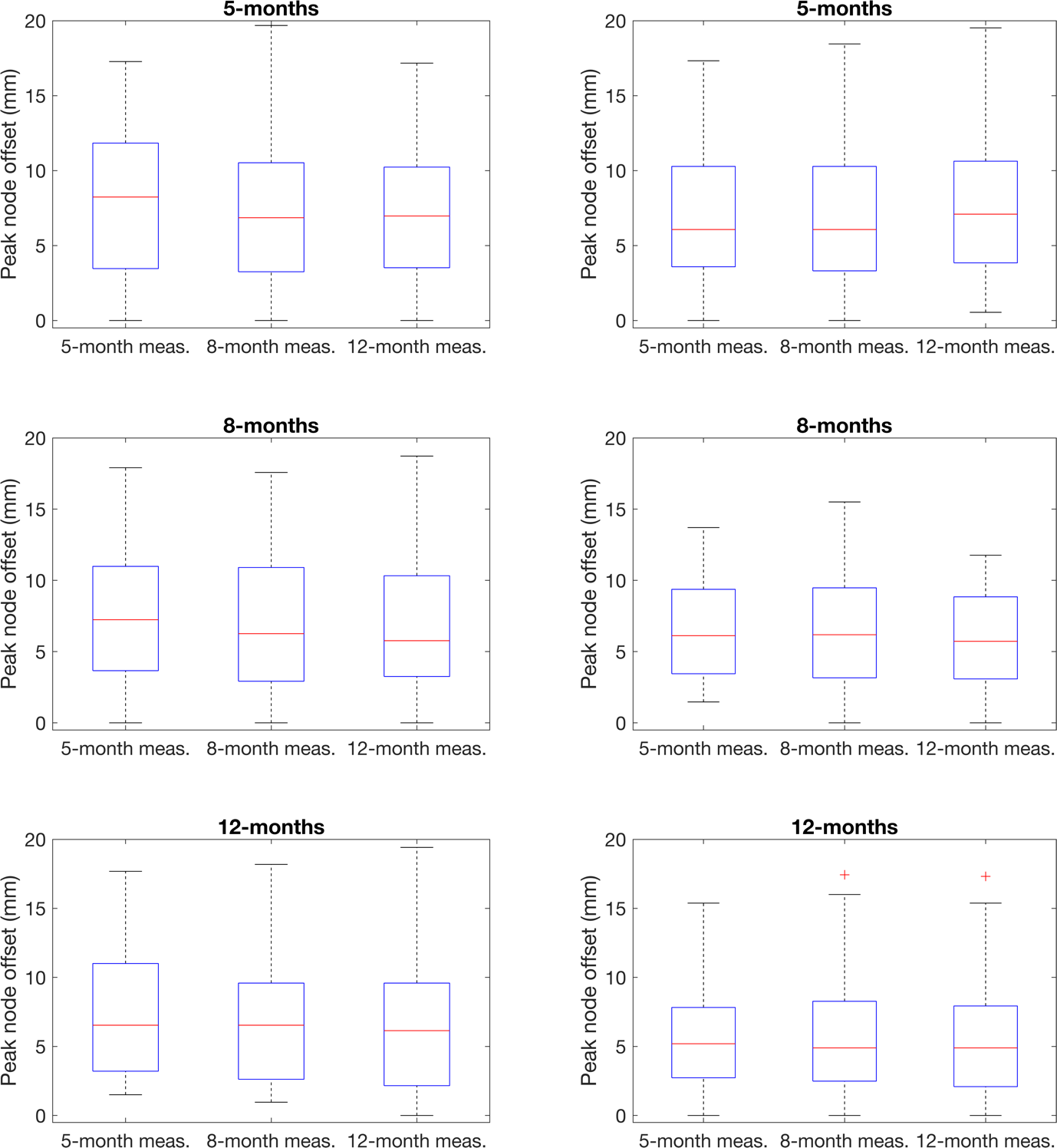
**

Supplementary figure 6: Individual-level peak node offset between the subject parameter pipeline and the constant parameter pipeline modified such that the head model size is determined by average head measurements at each of the three age points. There is no statistically significant difference between peak node offset between any of these distributions for a given hemisphere at a given age.
